# Transcriptome-based molecular systematics: *Rhodnius montenegrensis* (Triatominae) and its position within the *Rhodnius prolixus*–*Rhodnius robustus* cryptic–species complex

**DOI:** 10.1186/s13071-019-3558-9

**Published:** 2019-06-17

**Authors:** Raíssa N. Brito, Juliana A. Geraldo, Fernando A. Monteiro, Cristiano Lazoski, Rita C. M. Souza, Fernando Abad-Franch

**Affiliations:** 1 Grupo Triatomíneos, Instituto René Rachou, Fiocruz Minas Gerais, Fundação Oswaldo Cruz (Fiocruz), Belo Horizonte, Brazil; 2 Programa Interunidades de Pós-Graduação em Bioinformática, Universidade Federal de Minas Gerais, Belo Horizonte, Brazil; 3 Instituto René Rachou, Fiocruz Minas Gerais, Fundação Oswaldo Cruz (Fiocruz), Belo Horizonte, Brazil; 4 Laboratório de Epidemiologia e Sistemática Molecular, Instituto Oswaldo Cruz, Fundação Oswaldo Cruz (Fiocruz), Rio de Janeiro, Brazil; 5 Instituto de Biologia, Universidade Federal do Rio de Janeiro, Rio de Janeiro, Brazil; 6 Programa de Pós-Graduação em Medicina Tropical, Núcleo de Medicina Tropical, Faculdade de Medicina, Universidade de Brasília, Brasilia, Brazil

**Keywords:** *Rhodnius*, Triatominae, Molecular systematics, Transcriptomics

## Abstract

**Background:**

*Rhodnius montenegrensis* (Triatominae), a potential vector of Chagas disease, was described after *R. robustus*-like bugs from southwestern Amazonia. Mitochondrial *cytb* sequence near-identity with sympatric *R. robustus* (genotype II) raised doubts about the taxonomic status of *R. montenegrensis*, but comparative studies have reported fairly clear morphological and genetic differences between *R. montenegrensis* and laboratory stocks identified as *R. robustus*. Here, we use a transcriptome-based approach to investigate this apparent paradox.

**Results:**

We retrieved publicly-available transcriptome sequence-reads from *R. montenegrensis* and from the *R. robustus* stocks used as the taxonomic benchmark in comparative studies. We (i) aligned transcriptome sequence-reads to mitochondrial (*cytb*) and nuclear (ITS2, D2-*28S* and *AmpG*) query sequences (47 overall) from members of the *R. prolixus*–*R. robustus* cryptic–species complex and related taxa; (ii) computed breadth- and depth-coverage for the 259 consensus sequences generated by these alignments; and, for each locus, (iii) appraised query sequences and full-breadth-coverage consensus sequences in terms of nucleotide-sequence polymorphism and phylogenetic relations. We found evidence confirming that *R. montenegrensis* and *R. robustus* genotype II are genetically indistinguishable and, hence, implying that they are, in all likelihood, the same species. Furthermore, we found compelling genetic evidence that the benchmark ‘*R. robustus*’ stocks used in *R. montenegrensis* description and in later transcriptome-based comparisons are in fact *R. prolixus*, although likely mixed to some degree with *R. robustus* (probably genotype II, a.k.a. *R. montenegrensis*).

**Conclusions:**

We illustrate how public-domain genetic/transcriptomic data can help address challenging issues in disease-vector systematics. In our case-study, taxonomic confusion apparently stemmed from the misinterpretation of sequence-data analyses and misidentification of taxonomic-benchmark stocks. More generally, and together with previous reports of mixed and/or misidentified *Rhodnius* spp. laboratory colonies, our results call into question the conclusions of many studies (on morphology, genetics, physiology, behavior, bionomics or interactions with microorganisms including trypanosomes) based on non-genotyped ‘*R. prolixus*’ or ‘*R. robustus*’ stocks. Correct species identification is a prerequisite for investigating the factors that underlie the physiological, behavioral or ecological differences between primary domestic vectors of Chagas disease, such as *R. prolixus*, and their sylvatic, medically less-relevant relatives such as *R. robustus* (*s.l.*) including *R. montenegrensis*.

**Electronic supplementary material:**

The online version of this article (10.1186/s13071-019-3558-9) contains supplementary material, which is available to authorized users.

## Background

*Rhodnius prolixus* (Triatominae) is a primary vector of Chagas disease across northern South America, where infestation of rural houses by this species is common [1, 2]; it belongs to a group of closely-related taxa with nearly identical morphologies, i.e. the ‘*R. prolixus*–*R. robustus* cryptic–species complex’ [3–6]. Except for *R. prolixus*, the species in this complex (*R. robustus* (*s.l.*) [5, 6], *R. montenegrensis* [7] and *R. marabaensis* [8]) do not infest houses and have relatively little medical relevance [4, 9–11]. Why just one species within this group of close-kin bugs has the ability to stably infest houses is still unclear [6, 9, 11]. At least in part, this knowledge gap stems from the taxonomic uncertainty inherent to studying cryptic taxa. Thus, even though molecular systematics has contributed substantially to clarify the composition of the *R. prolixus*–*R. robustus* complex and the relations among its members, some controversies remain [3–6]. Using *R. montenegrensis* as a case-study, here we describe an approach that combines public-domain genetic/transcriptomic data and bioinformatics to address such controversies.

*Rhodnius montenegrensis* was described in 2012 by researchers of the Universidade Estadual Paulista ‘Júlio de Mesquita Filho’ (UNESP), Brazil, based on bugs resembling *R. robustus* [7]. The material used in this description came from UNESP colony ‘CTA 88’, which was founded with eight bugs collected in 2008 from *Attalea* palms in the southwestern Brazilian Amazon [7]. These bugs were compared to benchmark material identified as *R. robustus* from four laboratory colonies (‘CTA 83’ to ‘CTA 86’) founded with bugs collected in Peru and kept at UNESP since the early 1970s [7]. The original description included a 369-bp DNA sequence of *R. montenegrensis*’ mitochondrial cytochrome b (*cytb*) gene (GenBank ID: KR072682.1); a phylogenetic analysis recovered KR072682.1 as very closely related to a sequence of undisclosed origin or GenBank ID but labeled as ‘*R. robustus*’ (see figure 15 in [7]). In addition, the endonuclease *Bst*UI did not cleave nuclear rDNA 5.8S/ITS2 amplicons from putative *R. montenegrensis*, but cleaved at one site amplicons from the benchmark *R. robustus* colony bugs, thus implying that the 5.8S/ITS2 sequences of *R. montenegrensis* and *R. robustus* differ by at least one base at the enzyme’s restriction site [7]. The authors concluded that, although closely related, *R. montenegrensis* and *R. robustus* are morphologically and genetically distinct [7]. These findings received further support from a comparative-transcriptomics study showing that bugs identified as *R. montenegrensis* and bugs from UNESP’s *R. robustus* colony ‘CTA 85’, had “… a substantial quantity of fixed interspecific polymorphisms …”; this was interpreted as “… suggest[ing] a high degree of genetic divergence between the two species [that] likely corroborates the species status of *R. montenegrensis*” ([12], Abstract; see also [13] for details).

The striking similarity of *R. montenegrensis* and *R. robustus cytb* sequences was recently confirmed by a broader analysis [6] showing that *R. montenegrensis*’ KR072682.1 is nearly identical to *cytb* sequences from *R. robustus* genotype II, one of the *R. robustus* cryptic taxa identified by Monteiro et al. [5] in the early 2000s. While these results clearly suggest that *R. montenegrensis* is “… part of the variability of *R. robustus* II …” (caption of figure 2A in [6]), they raise the question of why morphology [7] and transcriptomics [12, 13] both discriminate *R. montenegrensis* from *R. robustus* bugs of Peruvian origin. This is even more intriguing when one considers that *R. robustus* II is the only *R. robustus* lineage known to occur in western-southwestern Amazonia [5, 6, 14–16]; *R. robustus* material from Peru, then, is expected to belong in genotype II and, hence, to be indistinguishable from *R. montenegrensis*.

**Fig. 1:**
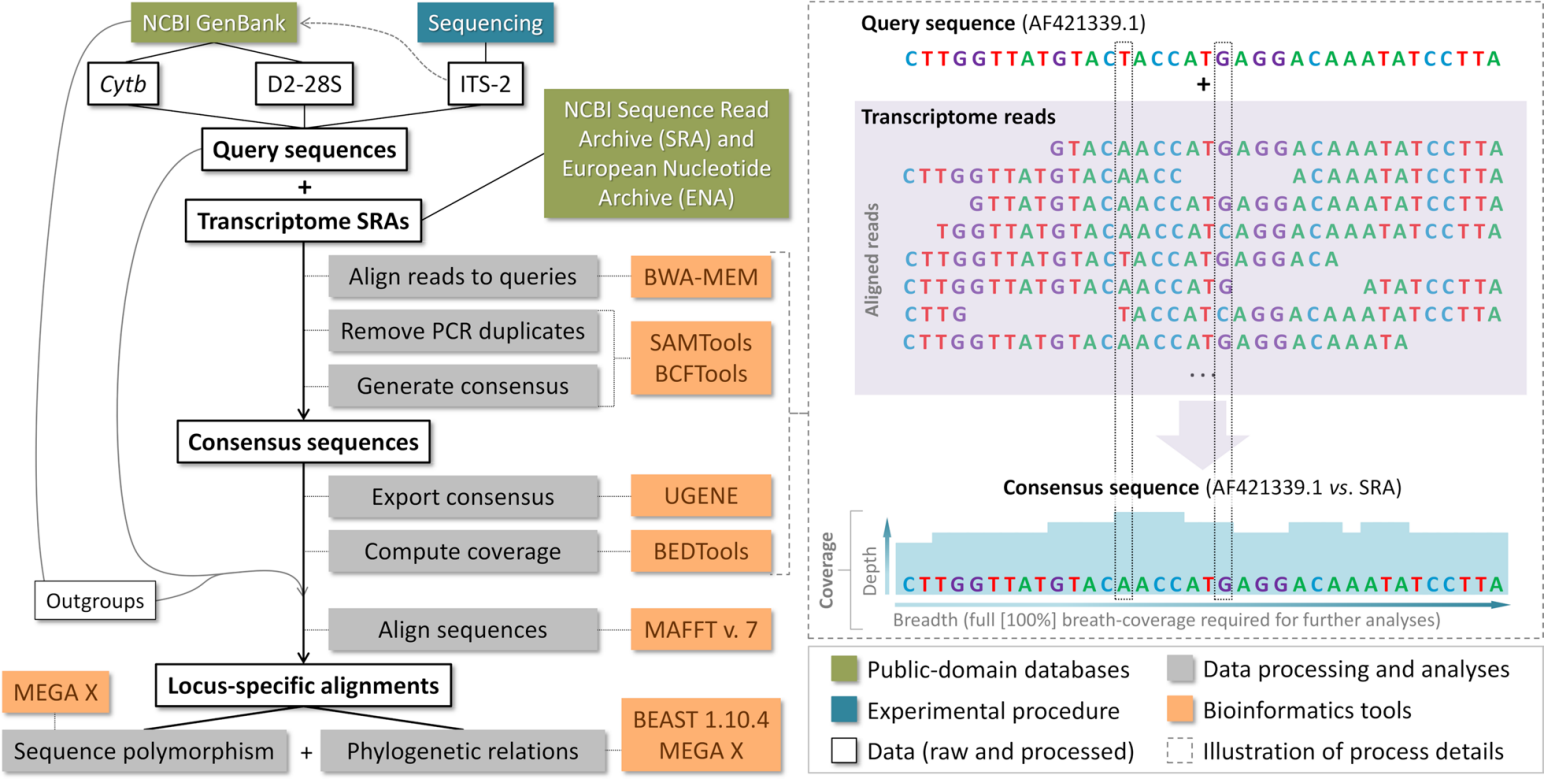
Transcriptome-based molecular systematics: a diagrammatic representation of our methodological approach. We determined 10 ITS2 query sequences and deposited them in NCBI’s (National Center for Biotechnology Information) GenBank (broken grey arrow). Other query and outgroup sequences were retrieved from GenBank, and transcriptome read archives (SRAs) from NCBI’s Sequence Read Archive and the European Nucleotide Archive (ENA). The right panel (broken-line box) illustrates some details of the process of generating consensus sequences from raw transcriptome reads plus query sequences and then computing breadth- and depth-coverage (pale-blue histograms behind the consensus sequence). The dotted-line vertical boxes highlight two variable sites: one in which the consensus sequence differs from the query (T/A), even though a minority of reads have T as in the query; and another in which a rare C variant appears in a minority of aligned reads and the consensus sequence, therefore, retains G as in the query

Recently, Monteiro and colleagues [6] suggested a possible explanation for these apparently contradictory findings. They observed (i) that the members of the *R. prolixus*–*R. robustus* species complex all have virtually identical phenotypes [1–4]; (ii) that several species-pairs within the complex are inter-fertile [17]; (iii) that there is evidence that many laboratory colonies of bugs identified as either *R. prolixus* or *R. robustus* (*s.l.*) are mixed/contaminated or wrongly labeled (see SI Appendix of [18]); and (iv) that *cytb* sequences of bugs from colonies labeled as ‘*R. robustus*’ from ‘Lima, Peru’ match *R. prolixus* sequences [19]. These observations suggest that the Peruvian *R. robustus* colonies kept at UNESP may have become contaminated with non-*R. robustus* material, with the main suspect being *R. prolixus* [6]. This hypothesis predicts that bugs drawn from the Peruvian ‘*R. robustus*’ colonies at UNESP will have *R. prolixus* genetic material, perhaps mixed to some degree with *R. robustus* (likely genotype II). In contrast, bugs from the younger (and hence less likely to have become contaminated) *R. montenegrensis* colonies should be genetically indistinguishable from *R. robustus* II. Here, we use publicly-available transcriptome data derived from UNESP *Rhodnius* spp. colonies to test these two predictions. More generally, we present a methodological approach (Fig. 1) that leverages public-domain information from traditional and next-generation sequencing projects to investigate the molecular systematics of cryptic species in the face of taxonomic confusion.

## Methods

### Transcriptome queries

We used selected DNA sequences from members of the *R. prolixus*–*R. robustus* cryptic–species complex to query publicly-available transcriptome sequence read archives (SRAs) derived from UNESP colonies, namely the *R. montenegrensis* colony and the Peruvian ‘*R. robustus*’ colonies used as the taxonomic benchmark in the description of *R. montenegrensis* [7] and in later transcriptome-based comparisons [12, 13] (Table 1). We chose three loci that have been widely used to study the systematics and evolution of *Rhodnius* spp. and other triatomines: the mitochondrial *cytb* [4–6, 11, 14, 16, 19] plus the nuclear rDNA ITS2 [6, 20] and D2-*28S* [5]. *Cytb* and D2-*28S* sequences were retrieved from GenBank, and our ten ITS2 query sequences (*R. prolixus* and *R. robustus* I to IV; GenBank: MK411269–MK411278) were determined using previously described primers and protocols [20]. Figure 1 presents a summary of our methods, and Additional file 1: Tables S1 and S2 provide details on query sequences including GenBank ID codes.

**Table 1:** Transcriptome raw data (sequence read archives) used in this study

Putative species	SRA	ENA run	Platform^a^	Colony	Material	Notes	Reference
*Rhodnius montenegrensis*	SRX1996481	SRR3995415	HiSeq 2500	UNESP ‘CTA 88’	Heads	Pool of 7 males	[12, 13]
	SRX1996482	SRR3995416	HiSeq 2500	UNESP ‘CTA 88’	Heads	Pool of 6 males	[12, 13]
*Rhodnius robustus*	SRX1996483	SRR3995417	HiSeq 2500	UNESP ‘CTA 85’	Heads	Pool of 6 males	[12, 13]
	SRX1996484	SRR3995418	HiSeq 2500	UNESP ‘CTA 85’	Heads	Pool of 6 males	[12, 13]
	ERX1387159	ERR1315266	HiSeq 2000	UNESP colony (Peru)	Rostrum + antennae	Pool of 21 females	Unpublished^b^
	ERX1387160	ERR1315268	HiSeq 2000	UNESP colony (Peru)	Rostrum + antennae	Pool of 19 males	Unpublished^b^

^a^Illumina, 2 × 100-bp paired-end reads

^b^CNRS (Centre National de la Recherche Scientifique, France) sequencing project; see [21]

*Abbreviations*: SRA, transcriptome access code at the Sequence Read Archive, National Center for Biotechnology Information (NCBI); ENA run, transcriptome run access code at the European Nucleotide Archive, European Molecular Biology Laboratory-European Bioinformatics Institute (EMBL-EBI); UNESP, Universidade Estadual Paulista ‘Júlio de Mesquita Filho’, Brazil

Figure 1 shows an outline of our methodological approach. We downloaded six public-domain transcriptome SRAs determined by two independent groups at UNESP and the French Centre National de la Recherche Scientifique (CNRS), respectively (see Table 1 and [12, 13, 21]). We aligned transcriptome paired-end reads to our query sequences using the Burrows-Wheeler aligner [22] with the BWA-MEM algorithm and default parameter values except for − t = 10, − B = 5, − O = 7.7, − L = 6.6, and − U = 18. We stored aligned reads in Binary Alignment Map (BAM) format, removed PCR duplicates with SAMtools markdup [23], and used SAMtools mpileup and BCFtools [23] to generate a consensus sequence for each alignment. We then exported consensus sequences in fasta format using UGENE [24], and computed sequence breadth-coverage and site-specific read depth-coverage using BEDTools [25]. We retained for further analyses consensus sequences with full-breadth-coverage of the query sequence (Fig. 1).

As a quality check for our focal results, we queried SRAs with (i) *cytb* and D2-*28S* sequences from *R. neglectus* and *R. nasutus*, which are relatively close kin to the members of the *R. prolixus*–*R. robustus* species complex [6]; and (ii) sequences of a putative nuclear intron (*AmpG*) from *R. prolixus* and *R. robustus* I–IV [15]. Our expectation was that these complementary queries would not generate any full-breadth-coverage consensus sequence.

### Sequence-polymorphism and phylogenetic analyses

We used MAFFT v.7 [26] to align, for each locus, query sequences, full-breadth-coverage consensus sequences and selected outgroup GenBank sequences (*R. barretti* JX273159.1 for *cytb*, *R. stali* AJ286890.2 for ITS2 and *R. nasutus* AF435856.1 for D2-*28S*); we made some manual adjustments to the ITS2 and D2-*28S* alignments (Additional file 2: Alignments S1–S3). Locus-specific alignments were first analyzed in terms of sequence polymorphism using MEGA X [27]. We computed a set of basic sequence diversity and similarity metrics (Additional file 1: Table S1), and examined segregating sites in detail to identify those that might be informative in the context of our research question – e.g., species-diagnostic character states and missense or nonsense mutations in the protein-coding *cytb* sequences. We used the bootstrap (1000 pseudo-replicates) to provide estimates of uncertainty for sequence diversity/similarity metrics. We then evaluated phylogenetic relations among the sequences in each alignment using the Bayesian approach implemented in BEAST v.1.10.4 [28]. For each locus, we completed four independent runs with Yule priors for 1,000,000 generations, sampling every 1000 steps and with a 25% burn-in. We then used FigTree v.1.4.4 (http://tree.bio.ed.ac.uk/software/figtree) to build maximum-credibility trees with the posterior probability cut-off set at 0.5. We assessed clade support based on posterior probabilities. We also estimated maximum-likelihood (ML) and maximum-parsimony (MP) trees in MEGA X [27]; the methods and results of these complementary analyses can be found in Additional file 3: Figure S1, Additional file 4: Figure S2, Additional file 5: Figure S3 and in the captions thereof. We finally examined the results of our sequence-polymorphism and phylogenetic analyses in the light of sequence depth-coverage, measured as the number of reads that aligned at each nucleotide position (see Fig. 1). In particular, full-breath-coverage consensus sequences with mean depth-coverage < 10 reads/position were regarded as unreliable [29], and consensus sequences with mean depth-coverage ≥ 10 reads/position, but with ≥ 15% of positions supported by < 10 reads, as dubious (see Additional file 1: Table S1 and Additional file 6: Figure S4).

## Results

### General results

**Fig. 2:**
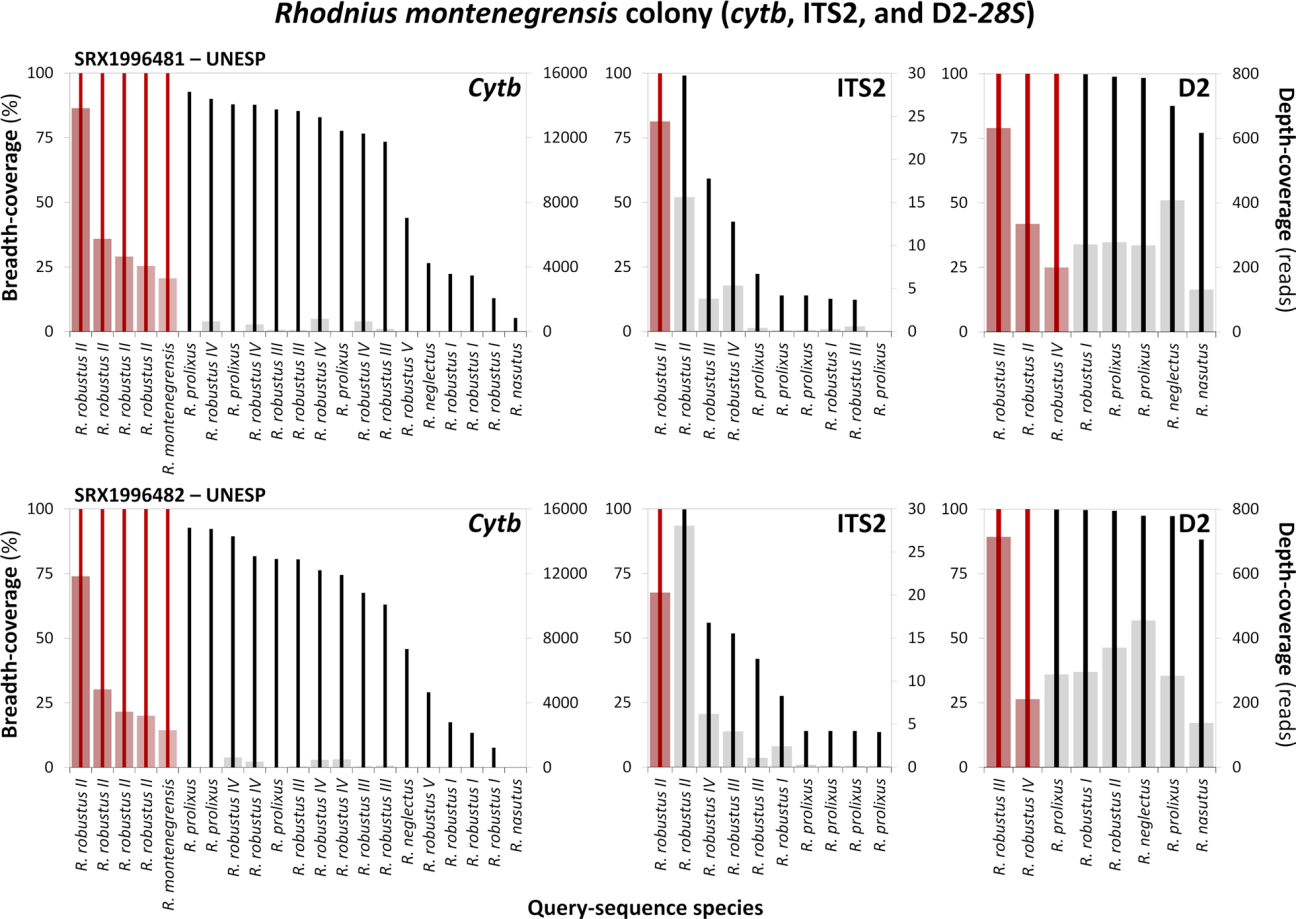
Alignment of *Rhodnius montenegrensis* transcriptome reads to *Rhodnius* spp. query sequences. The graphs present, for each of three loci (mitochondrial *cytb* plus nuclear rDNA ITS2, and D2-*28S*), breath-coverage (% of positions; bold vertical lines; left vertical axis) and mean depth-coverage (reads/position; bars; right vertical axis) for queries against two *R. montenegrensis* transcriptomes (SRX1996481 and SRX1996482; [7, 12, 13]). Red lines/bars highlight queries yielding full-breadth-coverage; queries yielding only partial-breadth-coverage are black/grey. *Abbreviation*: UNESP, Universidade Estadual Paulista ‘Júlio de Mesquita Filho’, Brazil

**Fig. 3:**
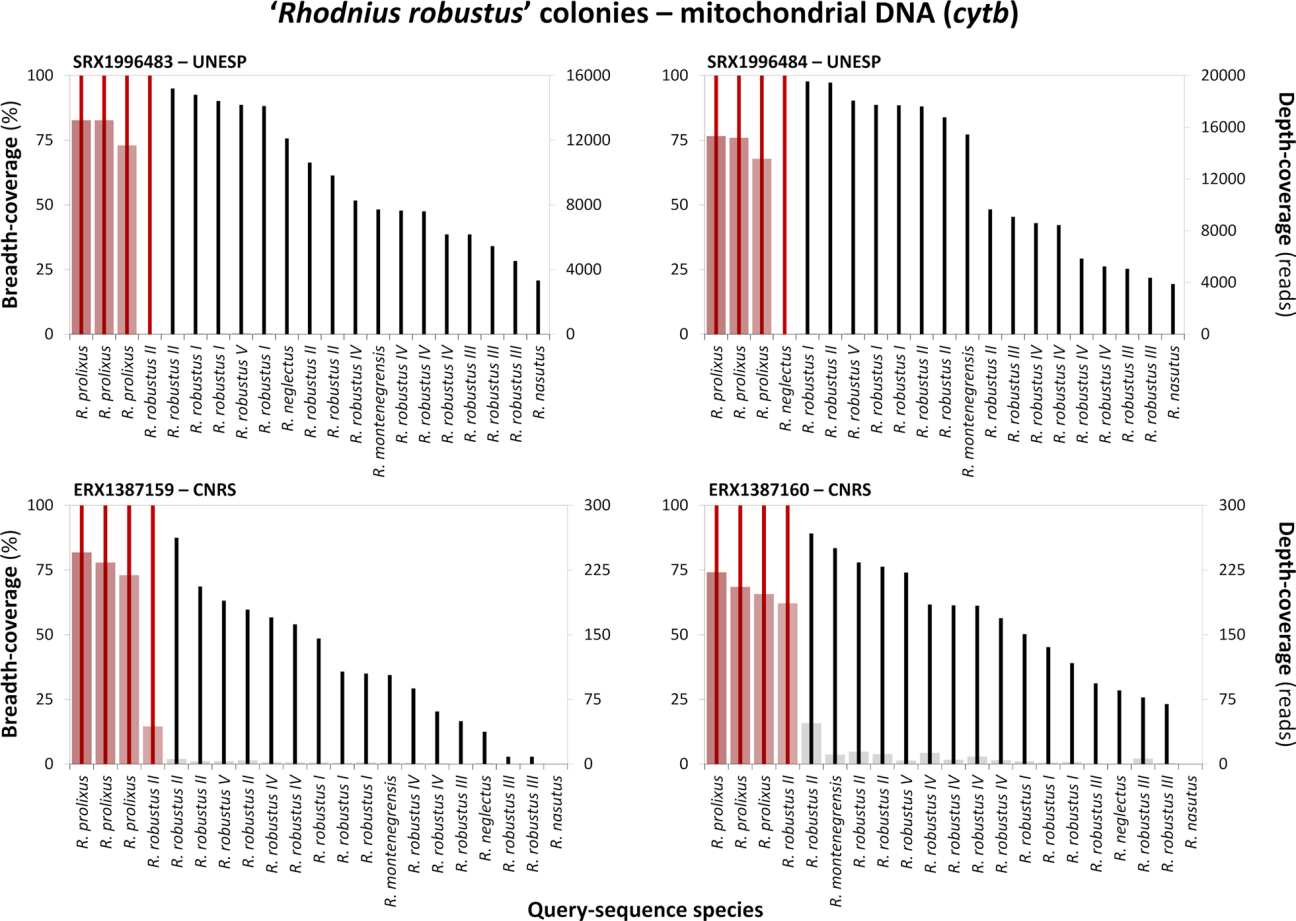
Alignment of *Rhodnius robustus* transcriptome reads to *Rhodnius* spp. *cytochrome b* sequences. Transcriptome reads were retrieved from the benchmark *Rhodnius robustus* stocks used in *R. montenegrensis*’ description and in later transcriptome-based comparisons. The graphs present breath-coverage (% of positions; bold vertical lines; left vertical axis) and mean depth-coverage (reads/position; bars; right vertical axis) for queries against four *R. robustus* transcriptomes (SRX1996483, SRX1996484, ERX1387159 and ERX1387160; [7, 12, 13, 21]). Red lines/bars highlight queries yielding full-breadth-coverage; queries yielding only partial-breadth-coverage are black/grey. *Abbreviations*: UNESP, Universidade Estadual Paulista ‘Júlio de Mesquita Filho’, Brazil; CNRS, Centre National de la Recherche Scientifique, France

**Fig. 4:**
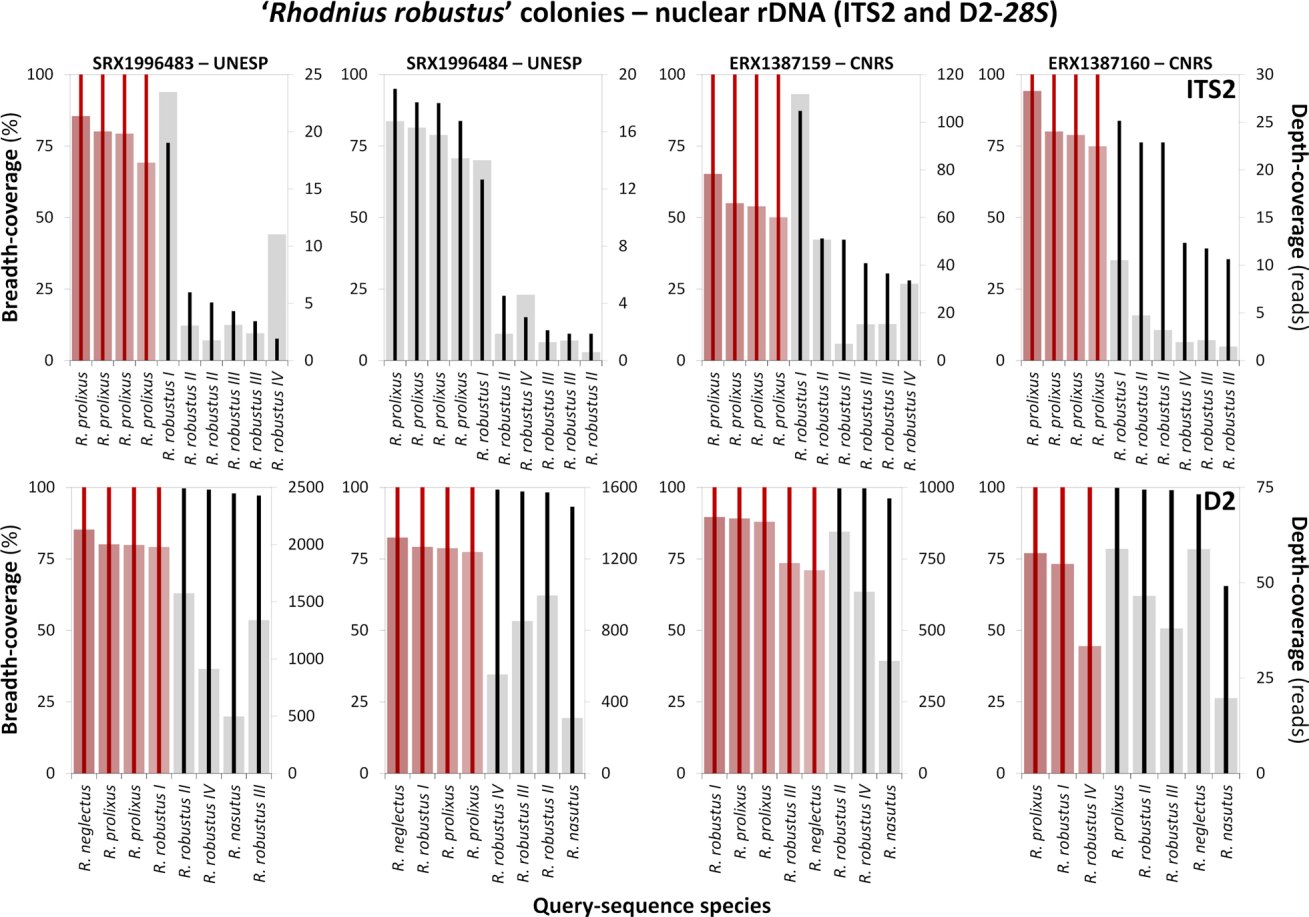
Alignment of *Rhodnius robustus* transcriptome reads to *Rhodnius* spp. ribosomal DNA sequences. Transcriptome reads were retrieved from the benchmark *Rhodnius robustus* stocks used in *R. montenegrensis*’ description and in later transcriptome-based comparisons; query sequences include two nuclear rDNA loci: ITS2 (upper row) and D2-*28S* (lower row). The graphs present breath-coverage (% of positions; bold vertical lines; left vertical axis) and mean depth-coverage (reads/position; bars; right vertical axis) for queries against four *R. robustus* transcriptomes (SRX1996483, SRX1996484, ERX1387159 and ERX1387160; [7, 12, 13, 21]). Red lines/bars highlight queries yielding full-breadth-coverage; queries yielding only partial-breadth-coverage are black/grey. *Abbreviations*: UNESP, Universidade Estadual Paulista ‘Júlio de Mesquita Filho’, Brazil; CNRS, Centre National de la Recherche Scientifique, France

We aligned sequence reads from six transcriptomes to query sequences representative of all (*cytb*) or all but one (ITS2 and D2-*28S*) known members of the *R. prolixus*–*R. robustus* cryptic–species complex; ITS2 and D2-*28S* sequences of the little-known *R. robustus* V are so far unavailable. Overall, our query dataset comprised 47 sequences (Additional file 1: Table S1). Using fairly stringent alignment/filter settings, we generated 61 full-breadth-coverage and 198 partial-breadth-coverage consensus sequences; no base aligned to the query sequence in 23 of our 282 queries (Additional file 1: Table S1). Depth-coverage was usually high for queries yielding full-breadth-coverage, but varied substantially across SRAs (Figs. 2, 3, 4, Tables 3, 4; see Additional file 1: Table S1 for breadth- and depth-coverage summary statistics across all queries, and Additional file 6: Figure S4 for full-breadth-coverage consensus sequences in which depth-coverage was < 10 reads at one or more positions.

### Mitochondrial *cytochrome b*

The 369-bp *R. montenegrensis cytb* sequence reported in the species’ description (KR072682.1) [7] differs at just one to four bases from those of bugs identified as *R. robustus* II collected in Rondônia, Brazil (including Monte Negro, the type locality of *R. montenegrensis*), and at five bases from that of a bug collected in Napo, Ecuador [5, 14, 30] (Table 2). We note that all *Rhodnius* spp. *cytb* sequences known to us have A at site 280 in our 663-bp alignment; the only exception seems to be EF071583.1 (*R. robustus* II from Rondônia [30]), which has G, a mutation that yields an Asparagine/Glycine change in the predicted protein (Additional file 2: Alignment S1). We strongly suspect that this first-codon position ‘mutation’ is a base-call error in EF071583.1. In support of this suspicion, we found that depth-coverage was lowest around position 280 when we aligned *R. montenegrensis* reads (SRAs SRX1996481 and SRX1996482) to query EF071583.1, with values of just 1.4–1.5% of the mean depth values (Table 3).

**Table 2:** Negligible mitochondrial *cytochrome b* sequence divergence between *Rhodnius montenegrensis* and *R. robustus* genotype II

Sequence		Geography			Divergence from *Rhodnius montenegrensis* KR072682.1 [7]				
GenBank ID	Reference	Locality	State/Province	Country	No. of bases	p-distance	SE	T3p+γ distance	SE
EF071583.1	[30]	Not reported	Rondônia	Brazil	1^a^	0.00271	0.00258	0.00276	0.00263
EF011720.1	[5]	Porto Velho	Rondônia	Brazil	2	0.00542	0.00383	0.00563	0.00429
EF011724.1	[14]	Monte Negro	Rondônia	Brazil	3^b^	0.00813	0.00427	0.00862	0.00528
AF421341.1	[5]	Not reported	Napo	Ecuador	5	0.01355	0.00524	0.01494	0.00732

*Notes*: The comparisons involve sequence KR072682.1 (from *Rhodnius montenegrensis*’ original description [7]) *vs R. robustus* II sequences from the type locality (Monte Negro), from the same sub-region (state of Rondônia, Brazil), and from a ~2000-km distant area (province of Napo, Ecuador) within western Amazonia

^a^Likely a base-call error in EF071583.1

^b^Including one probable base-call error in EF011724.1

*Abbreviations*: p-distance, observed proportion of segregating sites; SE, standard error (from 1000 bootstrap pseudo-replicates); T3p+γ distance, corrected proportion of segregating sites estimated using the best-fit model of nucleotide substitution (Tamura three-parameter model with γ-distributed rates – five categories, γ = 0.20)

**Table 3:** Full-breadth-coverage consensus sequences generated from transcriptomes determined using UNESP *Rhodnius montenegrensis* colonies

Transcriptome	Locus	Query		Depth-coverage			Identity	Divergence^a^	
NCBI SRA		Sequence	Species	Mean	Minimum	< 10 reads^b^ (%)	Percent	Distance	SE
SRX1996481	*cytb*	EF011724.1	*Rhodnius robustus* II	13,831.35	1996	–	99.85	0.00152	0.00144
		AF421341.1	*R. robustus* II	5745.50	48	–	99.55	0.00463	0.00252
		EF071583.1	*R. robustus* II	4646.05	67	–	99.70	0.00306	0.00205
		EF011720.1	*R. robustus* II	4061.95	25	–	99.85	0.00152	0.00140
		KR072682.1	*R. montenegrensis*	3298.10	12	–	100.00	0.00000	0.00000
	ITS2^c^	MK411275	*R. robustus* II	24.41	3	1.6	99.59	0.00411	0.00231
	D2-*28S*	AF435857.1	*R. robustus* III	631.85	18	–	100.00	0.00000	0.00000
		AF435858.1	*R. robustus* II	334.82	2	1.3	100.00	0.00000	0.00000
		AF435859.1	*R. robustus* IV	199.84	1	33.0	99.68	0.00316	0.00220
SRX1996482	*cytb*	EF011724.1	*R. robustus* II	11,834.29	1689	–	99.85	0.00152	0.00144
		AF421341.1	*R. robustus* II	4834.87	32	–	99.55	0.00463	0.00252
		EF071583.1	*R. robustus* II	3450.87	47	–	99.70	0.00306	0.00205
		EF011720.1	*R. robustus* II	3203.49	13	–	100.00	0.00000	0.00000
		KR072682.1	*R. montenegrensis*	2311.50	6	0.3	100.00	0.00000	0.00000
	ITS2^c^	MK411274	*R. robustus* II	20.29	1	29.6	99.32	0.00686	0.00290
	D2-*28S*	AF435857.1	*R. robustus* III	714.15	20	–	100.00	0.00000	0.00000
		AF435859.1	*R. robustus* IV	211.15	1	31.3	99.68	0.00316	0.00205

*Notes*: Depth-coverage (reads/position) and sequence identity (percent of identical bases) and divergence (model-based genetic distance estimates) of query *vs* consensus sequences are presented for three loci and two transcriptomes determined by UNESP researchers [12, 13]

^a^From the best-fit models of nucleotide substitution: Tamura three-parameter with γ-distributed rates for *cytb* (γ = 0.20) and D2-*28S* (γ = 0.05), and Tamura three-parameter for ITS2; standard errors (SE) computed after 1000 bootstrap pseudo-replicates

^b^Percent of positions at which depth-coverage was < 10 reads (Additional file 6: Figure S4)

^c^See details in Additional file 1: Table S2

*Abbreviations*: UNESP, Universidade Estadual Paulista ‘Júlio de Mesquita Filho’, Brazil; NCBI, National Center for Biotechnology Information, USA; SRA, transcriptome access code at NCBI’s Sequence Read Archive; *cytb*, mitochondrial *cytochrome b* gene; ITS2, second internal transcribed spacer of the nuclear ribosomal DNA; D2-*28S*, D2 variable region of the nuclear ribosomal DNA

When we used *R. montenegrensis*’ KR072682.1 as the query sequence, *R. montenegrensis* SRAs yielded consensus sequences with full-breadth-coverage and substantial depth-coverage (Table 3, Fig. 2, Additional file 1: Table S1). This was also the case, however, when we aligned the same two SRAs to our three *R. robustus* II *cytb* query sequences, with, in addition, consistently improved depth-coverage (Table 3, Fig. 2, Additional file 1: Table S1). Reads from the *R. montenegrensis* SRAs aligned with full-breadth-coverage and substantial depth-coverage to query EF011724.1 (*R. robustus* II from Monte Negro [14]) (Table 3, Fig. 2, Additional file 1: Table S1). These two consensus sequences were identical, and both differed at a single, second-codon position (A/T, with depth-coverage > 11,000 reads) from the query sequence. Because all *Rhodnius* spp. *cytb* sequences we are aware of, except for *R. pictipes*, have T at this position (623 in our 663-bp alignment; Additional file 2: Alignment S1), we also suspect a base-call error in EF011724.1. This query sequence, as well as the consensus sequences it generated from the two *R. montenegrensis* SRAs, differed at three third-codon positions from the KR072682.1 sequence used to support *R. montenegrensis* as a valid species [7] (see Additional file 2: Alignment S1). Figure 3 shows that transcriptome reads from UNESP ‘*R. robustus*’ colonies aligned to *R. prolixus* query sequences with the highest breadth- and depth-coverage.

**Fig. 5:**
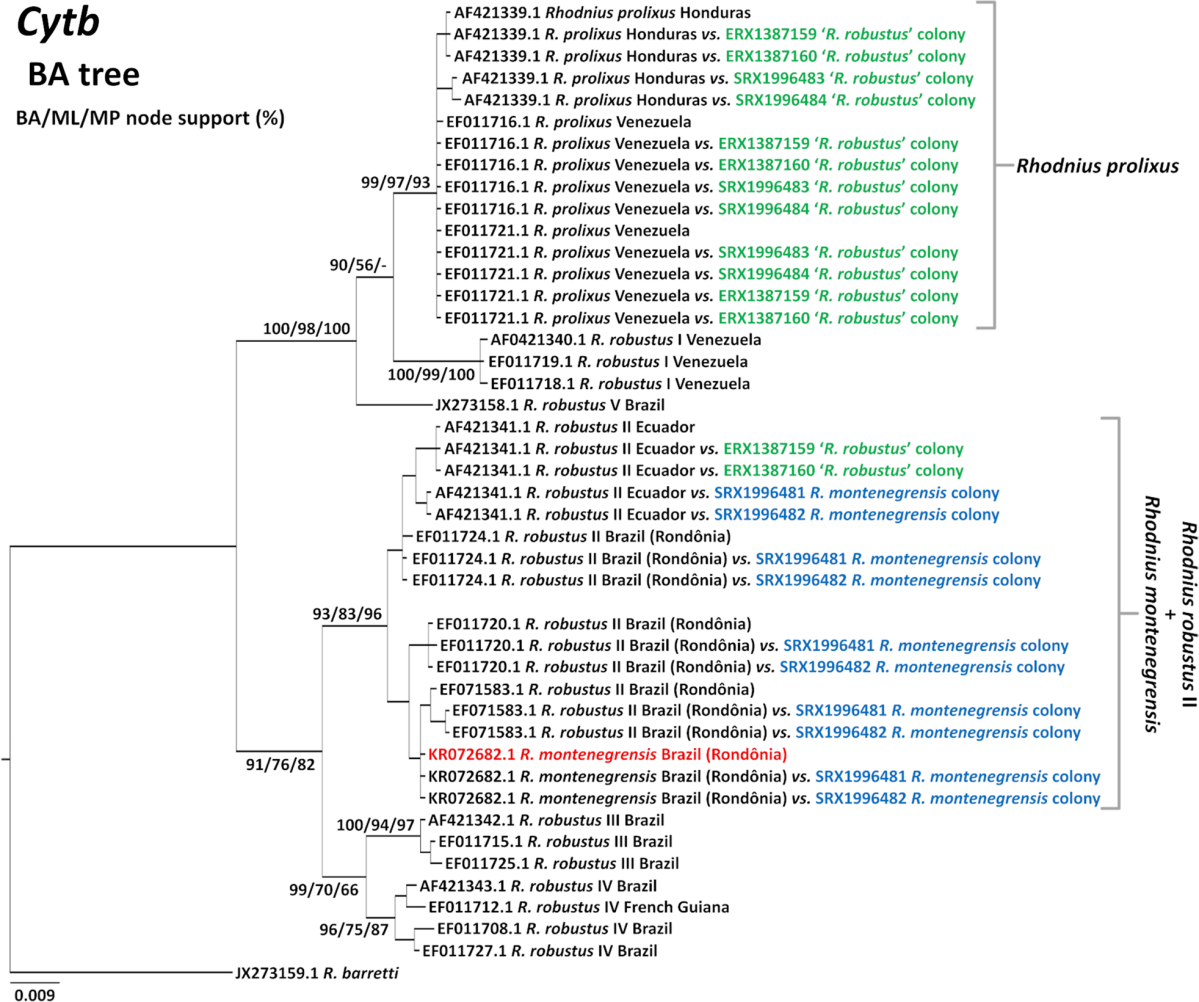
Mitochondrial *cytb* Bayesian phylogenetic tree of members of the *Rhodnius prolixus*–*R. robustus* cryptic–species complex. The tree was inferred using query sequences (black font) and the consensus sequences generated from *R. montenegrensis* (black/blue font) and *R. robustus* (black/green font) transcriptome-read archives (for which taxon labels include NCBI Sequence Read Archive codes). *Rhodnius montenegrensis*’ original sequence (KR072682.1; [7]) is highlighted in red font. Two full-breadth-coverage consensus sequences had mean depth-coverage < 10 reads/position and were therefore excluded from these analyses (see Table 4 and Additional file 6: Figure S4). Numbers at nodes are posterior probabilities from Bayesian analysis (BA) and bootstrap support values (1000 pseudo-replicates) for maximum-likelihood (ML) and maximum-parsimony (MP) topologies (see Additional file 3: Figure S1). The scale-bar indicates substitutions per site (from BA)

All the sequences in our *cytb* alignment, including consensus sequences generated from SRAs, comprise an open reading frame (Additional file 2: Alignment S1); this suggests that pseudogene sequences were absent from the dataset. Figure 5 shows the Bayesian *cytb* phylogenetic tree. This analysis recovered a well-supported clade including (i) *R. montenegrensis*’ original sequence (red in Fig. 5); (ii) all previously determined *R. robustus* II sequences; and (iii) full-breadth-coverage consensus sequences generated using *R. montenegrensis* or *R. robustus* II query sequences, irrespective of whether the SRAs were determined from UNESP colonies identified as *R. montenegrensis* or *R. robustus* (Fig. 5). ML and MP analyses also recovered this clade with moderate to high support (Fig. 5 and Additional file 3: Figure S1). These *cytb* trees also show that *R. prolixus* query sequences and the full-breadth-coverage consensus sequences they generated from ‘*R. robustus*’ transcriptome reads were almost identical; overall nucleotide diversity was π = 0.0020 ± 0.0011 SE, with maximum divergence of just ~ 0.3% (Table 4). Finally, the alignment of ‘*R. robustus*’ reads to the *R. robustus* II query AF421341.1 yielded two full-breadth-coverage consensus sequences that were identical to the query (Figs. 3, 5; Table 4).

**Table 4:** Full-breadth-coverage consensus sequences generated from transcriptomes determined using UNESP ‘*Rhodnius robustus*’ colonies

Transcriptome		Colony at UNESP	Locus	Query		Depth-coverage			Identity	Divergence^a^	
Group [refs.]	NCBI SRA			Sequence	Species	Mean	Minimum	< 10 reads^b^ (%)	Percent	Distance	SE
UNESP [12, 13]	SRX1996483	*Rhodnius robustus* ‘CTA 85’	*cytb*	EF011721.1	*R. prolixus*	13,238.94	7978	–	99.85	0.00152	0.00145
				EF011716.1	*R. prolixus*	13,235.83	8029	–	99.85	0.00152	0.00144
				AF421339.1	*R. prolixus*	11,680.80	29	–	99.70	0.00308	0.00211
				EF011724.1	*R. robustus* II^c^	6.18	2	90.2	99.85	0.00152	0.00144
			ITS2^d^	MK411270	*R. prolixus*	21.37	2	42.2	100.00	0.00000	0.00000
				MK411269	*R. prolixus*	20.03	2	36.9	100.00	0.00000	0.00000
				MK411271	*R. prolixus*	19.84	1	25.1	100.00	0.00000	0.00000
				MK411272	*R. prolixus*	17.30	2	53.7	100.00	0.00000	0.00000
			D2-*28S*	JQ897670.1^e^	*R. neglectus*	2131.15	8	1.0	99.34	0.00702	0.00407
				AF435862.1	*R. prolixus*	2002.57	315	–	100.00	0.00000	0.00000
				AF435860.1	*R. prolixus*	1996.21	313	–	100.00	0.00000	0.00000
				AF435861.1	*R. robustus* I	1978.62	316	–	100.00	0.00000	0.00000
	SRX1996484	*Rhodnius robustus* ‘CTA 85’	*cytb*	EF011721.1	*R. prolixus*	15,319.04	9426	–	99.85	0.00152	0.00145
				EF011716.1	*R. prolixus*	15,185.29	9224	–	99.85	0.00152	0.00144
				AF421339.1	*R. prolixus*	13,564.95	29	–	99.70	0.00306	0.00207
				JX273156.1	*R. neglectus* ^c^	8.63	1	53.5	99.40	0.00621	0.00314
			D2-*28S*	JQ897670.1^e^	*R. neglectus*	1319.28	6	5.7	99.68	0.00334	0.00257
				AF435861.1	*R. robustus* I	1266.95	169	–	100.00	0.00000	0.00000
				AF435862.1	*R. prolixus*	1259.78	173	–	100.00	0.00000	0.00000
				AF435860.1	*R. prolixus*	1238.28	188	–	100.00	0.00000	0.00000
CNRS [21]	ERX1387159	*Rhodnius robustus* ‘Peru’	*cytb*	EF011721.1	*R. prolixus*	245.46	102	–	100.00	0.00000	0.00000
				AF421339.1	*R. prolixus*	233.66	1	2.7	100.00	0.00000	0.00000
				EF011716.1	*R. prolixus*	219.03	60	–	99.85	0.00152	0.00144
				AF421341.1	*R. robustus* II	43.65	6	10.9	100.00	0.00000	0.00000
			ITS2^d^	MK411271	*R. prolixus*	78.31	21	–	100.00	0.00000	0.00000
				MK411272	*R. prolixus*	66.01	20	–	100.00	0.00000	0.00000
				MK411270	*R. prolixus*	64.70	18	–	100.00	0.00000	0.00000
				MK411269	*R. prolixus*	60.10	15	–	99.87	0.00139	0.00130
			D2-*28S*	AF435861.1	*R. robustus* I	896.28	28	–	100.00	0.00000	0.00000
				AF435860.1	*R. prolixus*	891.44	34	–	100.00	0.00000	0.00000
				AF435862.1	*R. prolixus*	879.61	37	–	100.00	0.00000	0.00000
				AF435857.1	*R. robustus* III	735.23	2	2.1	99.84	0.00159	0.00154
				JQ897670.1^e^	*R. neglectus*	710.08	3	6.9	99.84	0.00165	0.00187
	ERX1387160	*Rhodnius robustus* ‘Peru’	*cytb*	EF011721.1	*R. prolixus*	222.43	136	–	100.00	0.00000	0.00000
				AF421339.1	*R. prolixus*	205.40	1	3.2	100.00	0.00000	0.00000
				EF011716.1	*R. prolixus*	197.09	60	–	99.85	0.00152	0.00144
				AF421341.1	*R. robustus* II	186.42	19	–	100.00	0.00000	0.00000
			ITS2^d^	MK411271	*R. prolixus*	28.28	9	2.1	100.00	0.00000	0.00000
				MK411269	*R. prolixus*	24.03	7	6.9	100.00	0.00000	0.00000
				MK411272	*R. prolixus*	23.66	5	14.4	100.00	0.00000	0.00000
				MK411270	*R. prolixus*	22.48	5	8.6	100.00	0.00000	0.00000
			D2-*28S*	AF435860.1	*R. prolixus*	57.74	3	1.6	100.00	0.00000	0.00000
				AF435861.1	*R. robustus* I	54.91	3	0.5	100.00	0.00000	0.00000
				AF435859.1	*R. robustus* IV	33.38	1	34.3	100.00	0.00000	0.00000

*Notes*: Depth-coverage (reads/position) and sequence identity (percent of identical bases) and divergence (model-based genetic distance estimates) of query *vs* consensus sequences are presented for three loci and four transcriptomes determined by two research groups

^a^From the best-fit models of nucleotide substitution: Tamura three-parameter with γ-distributed rates for *cytb* (γ = 0.20) and D2-*28S* (γ = 0.05), and Tamura three-parameter for ITS2; standard errors (SE) computed after 1000 bootstrap pseudo-replicates

^b^Percent of positions at which depth-coverage was < 10 reads (Additional file 6: Figure S4)

^c^Consensus sequences with mean depth-coverage < 10 reads/position (Additional file 1: Table S1, Additional file 6: Figure S4) that were excluded from phylogenetic analyses

^d^See details in Additional file 1: Table S2

^e^JQ897670.1 is from a bug identified as *Rhodnius neglectus* from ‘Orellana, Ecuador’ (see [31]), where neither *R. neglectus* nor *R. prolixus* occur (*R. robustus* II and *R. barretti* do); this is most likely a case of misidentification or mislabeling of the specimen

*Abbreviations*: UNESP, Universidade Estadual Paulista ‘Júlio de Mesquita Filho’, Brazil; CNRS, Centre National de la Recherche Scientifique, France; NCBI, National Center for Biotechnology Information, USA; SRA, transcriptome access code at NCBI’s Sequence Read Archive; *cytb*, mitochondrial *cytochrome b* gene; ITS2, second internal transcribed spacer of the nuclear ribosomal DNA; D2-*28S*, D2 variable region of the nuclear ribosomal DNA

From these *cytb* sequence-data analyses we conclude that *R. montenegrensis* is, in all likelihood, the *R. robustus* cryptic taxon dubbed ‘*R. robustus* II’ by Monteiro et al. [5] in 2003. In addition, we found evidence strongly suggesting that at least some of the UNESP ‘*R. robustus*’ colonies used as the taxonomic benchmark to infer that *R. montenegrensis* is distinct from *R. robustus* [7, 12, 13] contain large amounts of *R. prolixus* mitochondrial DNA, and perhaps also smaller amounts of mitochondrial DNA from *R. robustus* II (i.e. *R. montenegrensis*) (Figs. 3, 5, Table 4).

### Nuclear ribosomal ITS2

We generated 14 full-length consensus sequences from five of the six SRAs we queried with our 10 ITS2 *Rhodnius* spp. sequences (Tables 3, 4; Additional file 1: Tables S1, S2). Depth-coverage values were overall substantially smaller for ITS2 than for *cytb* transcriptome queries (Figs. 2, 3, 4 and Tables 3, 4; see also Additional file 1: Table S1 and Additional file 6: Figure S4). We generated two full-breadth-coverage consensus sequences when we aligned *R. montenegrensis* SRAs to ITS2 sequences from *R. robustus* II collected in Rondônia, Brazil (Fig. 2, Table 3). We identified six variable sites (π = 0.005 ± 0.002 SE) and three indels when we compared these four *R. robustus* II/*R. montenegrensis* sequences (Additional file 2: Alignment S2). The MK411275 (*R. robustus* II from Rondônia) *vs* SRX1996482 query generated a 99.86% breadth-coverage (732 out of 733 bp) consensus sequence with mean depth-coverage of 28.06 reads (Additional file 1: Table S1). In addition, queries performed with our four *R. prolixus* ITS2 sequences generated full-breadth-coverage consensus sequences in three of the four SRAs derived from UNESP ‘*R. robustus*’ colonies, although depth-coverage was low for those generated from SRX1996483 (Fig. 4, Table 4, Additional file 6: Figure S4). We found just two variable sites (π = 0.0007 ± 0.0006 SE) and no indels in the comparison of this subset of *R. prolixus* and ‘*R. robustus*’ query and consensus sequences (Additional file 2: Alignment S2).

**Fig. 6:**
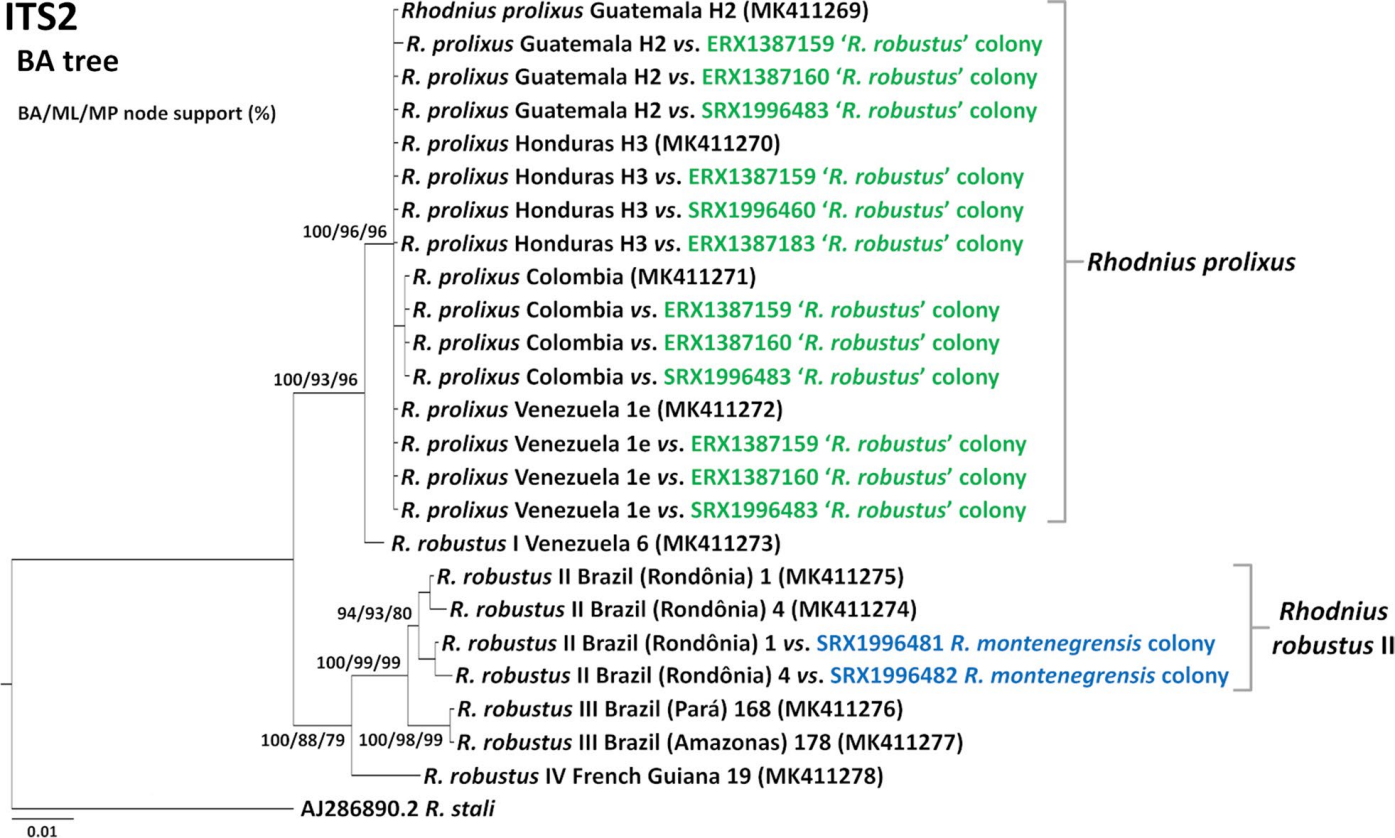
Nuclear ribosomal ITS2 Bayesian phylogenetic tree of members of the *Rhodnius prolixus*–*R. robustus* cryptic–species complex. The tree was inferred using query sequences [black font; new GenBank ID codes for ITS2 sequences generated for this study (MK411269–MK411278) are presented in parentheses] and the consensus sequences generated from *R. montenegrensis* (black/blue font) and *R. robustus* (black/green font) transcriptome-read archives (for which taxon labels include the query names and the NCBI Sequence Read Archive codes). Numbers at nodes posterior probabilities from Bayesian analysis (BA) and bootstrap support values (1000 pseudo-replicates) for maximum-likelihood (ML) and maximum-parsimony (MP) topologies (see Additional file 4: Figure S2). The scale-bar indicates substitutions per site (from BA)

Phylogenetic analyses recovered consistent ITS2 tree topologies (Fig. 6, Additional file 4: Figure S2). Full-breadth-coverage consensus sequences from *R. montenegrensis* SRAs and the *R. robustus* II query sequences used to generate them clustered together in a moderately- to well-supported clade. Support was higher for the clustering of *R. prolixus* query sequences with the full-breadth-coverage consensus sequences they generated from three ‘*R. robustus*’ SRAs (Fig. 6, Additional file 4: Figure S2).

We finally recall that, in the description of *R. montenegrensis*, da Rosa et al. [7] reported that *Bst*UI cleaved ‘*R. robustus*’ ITS2 at one site, but did not cleave *R. montenegrensis* ITS2 amplicons; here, we found that *Bst*UI’s restriction site (CGCG) is absent from the ITS2 sequences of *R. montenegrensis* and *R. robustus* II, III and IV, but present (sites 66–69 of our alignment; Additional file 2: Alignment S2) in those of *R. robustus* I and *R. prolixus*, including the latter species’ genome [18], with, e.g. query sequence MK411269 (*R. prolixus* from Guatemala; Additional file 1: Table S2) yielding a 100%-identity BLASTn hit in RproC3 assembly contig ACPB03046858.1 at VectorBase (https://www.vectorbase.org/). Thus, the *Bst*UI digestion results in [7] suggest that bugs from UNESP ‘*R. robustus*’ colonies have nuclear rDNA similar to that expected in bugs of the *R. prolixus*–*R. robustus* I clade.

Taken together, these ITS2 data lend clear support to the *cytb*-based findings described above. In particular, they (i) confirm that *R. montenegrensis* is genetically indistinguishable from *R. robustus* II, and (ii) further suggest that the UNESP ‘*R. robustus*’ colonies used as the taxonomic benchmark to conclude that *R. montenegrensis* is distinct from *R. robustus* [7, 12, 13] are mainly or fully composed of bugs with *R. prolixus* DNA, with no detectable *R. robustus* nuclear ITS2 sequences.

### Nuclear ribosomal D2-*28S*

Our D2-*28S R. prolixus*–*R. robustus* complex query sequences generated 21 full-breadth-coverage sequences from the six transcriptome SRAs; depth-coverage was overall substantial (typically > 250 reads/position on average) except for queries against SRA ERX1387160 (Figs. 2, 4; Tables 3, 4). Depth-coverage was, however, low over certain stretches of some full-breadth-coverage consensus sequences (Tables 3, 4; Additional file 6: Figure S4). When we used *R. robustus* IV’s AF435859.1 as the query, for example, mean depth-coverage was high for the consensus sequences generated from *R. montenegrensis*’ SRAs, yet depth-coverage fell to < 10 reads/position between positions ~ 260 and ~ 470 (Table 3, Additional file 6: Figure S4). Similarly, the AF435859.1 *vs* ERX1387160 query generated a full-breadth-coverage consensus sequence with 217 positions (34.3%) supported by < 10 reads each (Table 4, Additional file 6: Figure S4). We therefore regard the consensus sequences generated using our *R. robustus* IV query as dubious. Queries using the *R. robustus* III sequence generated full-breadth-coverage sequences from the two *R. montenegrensis* SRAs (with substantial depth-coverage; Fig. 2) and from one ‘*R. robustus*’ SRA (Fig. 4), for which depth-coverage was < 100 reads/position at 103 positions, including a stretch with < 10 reads/position between positions 539 and 551 (Table 4, Additional file 6: Figure S4). We note that position 540 in our 633-bp alignment (Additional file 2: Alignment S3) is the variable position that separates the clade including *R. robustus* II–IV (all with T) and that including *R. prolixus* and *R. robustus* I (with a derived C) [5]. We generated one full-length sequence using *R. robustus* II from Ecuador (AF435858.1) as the query (Fig. 2); depth-coverage was high except for a 70-position section with depth-coverage below 100 reads/position, including < 20 reads at positions 351–369 and < 10 reads at nine positions within this 19-bp stretch, which includes three variable positions (Table 3, Additional file 2: Alignment S3 and Additional file 6: Figure S4).

Our *R. prolixus* (AF435860.1 and AF435862.1) and *R. robustus* I (AF435861.1) D2-*28S* query sequences were identical and generated full-breadth-coverage consensus sequences from all ‘*R. robustus*’ SRAs (Fig. 4); the exception was *R. prolixus* query AF435862.1 *vs* ERX1387160, for which no read aligned to the last base (Table 4, Additional file 1: Table S1). These full-breadth-coverage consensus sequences did not differ by a single base from the also identical query sequences. Depth-coverage was overall high (except, as mentioned above, for SRA ERX1387160), with only a few, short stretches having low depth-coverage (Table 4, Additional file 6: Figure S4).

D2-*28S* sequences produced gene trees with relatively poor resolution; although the clustering of query and consensus sequences overall mirrored the patterns described for *cytb* and ITS2 (Figs. 5, 6), most tree nodes had low to very low support (Additional file 5: Figure S3). Overall, these D2-*28S* data further suggest that the benchmark UNESP’s ‘*R. robustus*’ colonies are mainly or fully composed of bugs with *R. prolixus* DNA, with only one *R. robustus* query (genotype IV) generating a full-breadth-coverage, yet low-depth-coverage, consensus sequence. The data were less informative with regard to the relation between *R. montenegrensis* and *R. robustus* II, likely because the only *R. robustus* II D2-*28S* sequence so far available (AF435858.1) is from an Ecuadorian bug caught ~ 2000 km from *R. montenegrensis*’ type locality. Our finding that reads from three ‘*R. robustus*’ SRAs aligned with full-breadth- and high depth-coverage to one *R. neglectus* query (Fig. 4, Table 4) is discussed in the next sub-section.

### Quality check: *cytb*, D2-*28S* and *AmpG*

As expected, no full-breadth-coverage sequences were generated when we aligned our study SRAs to *R. nasutus* sequences or to *AmpG* sequences from members of the *R. prolixus*–*R. robustus* species complex (Additional file 1: Table S1). *Rhodnius neglectus* queries, however, generated four full-breadth-coverage consensus sequences (Figs. 3, 4, Table 4). One *R. neglectus cytb* query (JX273156.1) yielded full-breadth-coverage against one ‘*R. robustus*’ SRA, yet with mean depth-coverage < 10 reads/position (Fig. 3, Table 4). In contrast, the only available *R. neglectus* D2-*28S* query [31] yielded both full-breadth-coverage and substantial mean depth-coverage (albeit with a ~ 45-bp stretch with depth-coverage < 20 reads/position) against three ‘*R. robustus*’ SRAs (Fig. 4, Table 4). This D2-*28S* sequence (JQ897670.1) is from a bug identified as *R. neglectus* but reportedly collected in western Amazonia (‘Orellana, Ecuadorʼ) [31], where *R. neglectus* does not occur [9, 10]. Therefore, this is most likely a case of misidentification or mislabeling of the specimen (voucher ‘UCR_ENT_00052203’ at the Entomology Collection of the University of California, Riverside) [31] (see Table 4).

## Discussion

In this report we have illustrated how publicly available transcriptome data can be used to clarify the systematics of a taxonomically challenging group of cryptic disease-vector species. This transcriptome-based approach to molecular systematics has, to our knowledge, not been used before in vector studies; it is overall analogous to the assembly of mitochondrial genes from transcriptome data used to study, for example, poison frogs [32], catfish [33], true bugs [34] or ants [35] (see also [36]). We found evidence confirming that *R. montenegrensis*, a species described in 2012 [7], is genetically indistinguishable from *R. robustus* II, one of the sibling taxa within *R. robustus* (*s.l.*) that Monteiro and colleagues discovered in 2003 [5, 6]. To solve the paradox that *R. montenegrensis* appears to be morphologically and genetically distinct from *R. robustus* [7, 12, 13], we then showed that the ‘*R. robustus*’ stocks used as the taxonomic benchmark in *R. montenegrensis*’ description [7] and in later transcriptome-based comparisons [12, 13] are almost certainly *R. prolixus*, likely mixed to some degree with *R. robustus*.

We note that our confirmation that *R. montenegrensis* and *R. robustus* II are almost identical genetically does not invalidate the former as a separate species – it just shows that ‘*Rhodnius montenegrensis*’ is the binomial for what we informally called ‘*Rhodnius robustus* II’ [5, 6]. Our results, in any case, provide an example of how triatomine-bug taxonomic research can be confounded when sequence-data analyses are loosely interpreted [6]. Thus, the *cytb* data presented in the description of *R. montenegrensis* [7] already showed that *R. montenegrensis* and *R. robustus* II are all but indistinguishable at that locus (Table 2). However, instead of pointing out the virtual identity of *R. montenegrensis* and *R. robustus* II sequences, da Rosa et al. [7] underscored the (effectively negligible) differences – a stance later mirrored in a study involving *cytb*-based identification of *R. montenegrensis* specimens [37]. As noted by Monteiro et al. [6], this is probably also the case for *R. marabaensis* and *R. robustus* III from southeastern Amazonia [5]; *R. marabaensis* sequences, however, have not been made available in public databases [8]. Along the same lines, the hypothesis that *R. milesi* [38] and *R. taquarussuensis* [39] are *R. neglectus* variants (see [6, 10]) recently received empirical support from molecular systematics [6, 40]. A further example of taxonomic uncertainty is *R. zeledoni* [41], whose holotype (the only known specimen) is strikingly similar to the sympatric *R. domesticus* [1, 6, 42]; however, the data needed to address this uncertainty are so far unavailable.

Our results also show how the use of mixed or misidentified *Rhodnius* spp. colonies can confound taxonomic research even further – and how we can take advantage of public-domain molecular data to clarify cryptic–species systematics in the face of such confusion. Perhaps more importantly, our finding that some putative ‘*R. robustus*’ colonies are fully or almost fully composed of bugs with *R. prolixus* DNA contributes to casting doubts over the conclusions of the many studies that made use of non-genotyped ‘*R. prolixus*’ or ‘*R. robustus*’ laboratory stocks. Mesquita et al. [18] noted this problem in their quest for a pure *R. prolixus* stock to be used in genome sequencing. Of the 15 putative *R. prolixus* colonies they genotyped, just four were pure: one had both mitochondrial and nuclear *R. robustus* II DNA and ten had introgressed *R. robustus* IV mitochondrial DNA (see p. 28 of Appendix SI of [18]). As transcriptome (and genome) data from other putative *R. prolixus* and *R. robustus* colonies progressively accrue, approaches analogous to the one we illustrate here may help elucidate their taxonomic identity and genetic integrity. This would be particularly interesting in the case of putative *R. prolixus* colonies derived from the legendary stock used by Sir Vincent B Wigglesworth in his seminal studies on insect physiology [43], but would also be valuable for assessing the taxonomic status of bugs used in more recent research on *Rhodnius* spp. morphology, genetics, physiology, behavior, bionomics or interactions with microorganisms including *Trypanosoma cruzi* (e.g. [44–48]). Similarly, our results suggest that the value of several approaches put forward to investigate the systematics of cryptic or near-cryptic *Rhodnius* taxa (including, for example, the use of quantitative phenotypic traits [7, 49] or ctyogenetics [39, 50]) will have to be reappraised after careful consideration of the specific status of the bugs, whether field-collected or laboratory-reared, used in comparative analyses.

## Conclusions

Here, we have illustrated how public-domain transcriptome reads and locus-specific sequences can be combined to address challenging issues in vector systematics. Using query sequences from mitochondrial and nuclear loci, six publicly-available raw transcriptome datasets, and a straightforward bioinformatics approach, we (i) confirmed that *R. montenegrensis* and *R. robustus* II are in all likelihood the same species, and (ii) showed that the UNESP ‘*R. robustus*’ stocks used as the taxonomic benchmark in *R. montenegrensis*’ description and in later comparative studies are most likely a mixture of (mainly) *R. prolixus* and (partly) *R. robustus* (probably genotype II). In this particular instance of taxonomic confusion, misinterpretation of sequence-data analyses was compounded by the misidentification of taxonomic-benchmark laboratory stocks. More generally, and together with previous reports of mixed and/or misidentified *Rhodnius* spp. colonies, our results call into question the conclusions of many studies based on non-genotyped ‘*R. prolixus*’ or ‘*R. robustus*’ stocks. *Rhodnius prolixus* and *R. robustus* (*s.l.*) are similar in many respects, but differ in a fundamental way: the former is a primary domestic vector of Chagas disease, whereas the latter comprises a suite of sylvatic species (including *R. montenegrensis*) of limited medical relevance. Correct species identification will be key to any attempt at understanding what physiological, behavioral or ecological factors may underlie this crucial difference.

## Additional files

**Additional file 1: Table S1.** Details on transcriptome-read queries. **Table S2.** ITS2 sequences generated in this study.

**Additional file 2: Alignment S1.** *Cytb* sequences. **Alignment S2.** ITS2 sequences. **Alignment S3.** D2-*28S* sequences.

**Additional file 3: Figure S1.** *Cytb* trees of members of the *Rhodnius prolixus-R. robustus* complex. Maximum-likelihood (ML; Tamura 3-parameter+γ) and maximum-parsimony (MP) trees using query sequences (black) and consensus sequences from *R. montenegrensis* (black/blue) and *R. robustus* (black/green) transcriptome-read archives (with NCBI codes). *Rhodnius montenegrensis*’ original sequence (KR072682.1; [7]) highlighted in red. Consensus sequences with mean depth-coverage <10 reads/position were excluded (Table 4, Additional file 6: Figure S4). Node-support: 1000 bootstrap pseudo-replicates. Scale-bars: substitutions/site (ML) and number of substitutions (MP).

**Additional file 4: Figure S2.** ITS2 trees of members of the *Rhodnius prolixus-R. robustus* complex. Maximum-likelihood (ML; Tamura three-parameter) and maximum-parsimony (MP) inferred using query sequences (black) and consensus sequences from *R. montenegrensis* (black/blue) and *R. robustus* (black/green) transcriptome-read archives (with NCBI codes). Node-support: 1000 bootstrap pseudo-replicates. Scale-bars: substitutions/site (ML) and number of substitutions (MP).

**Additional file 5: Figure S3.** D2-*28S* phylogenetic trees of members of the *Rhodnius prolixus-R. robustus* complex and related taxa. Bayesian analysis (BA), maximum-likelihood (ML; Tamura 3-parameter+γ) and maximum parsimony (MP) trees based on query sequences (black) and consensus sequences from *R. montenegrensis* and *R. robustus* transcriptome-read archives (with NCBI codes). JQ897670.1 is from a bug identified as ‘*Rhodnius neglectus*’ from ‘Orellana, Ecuador’ [31], where *R. neglectus* does not occur; most likely misidentification/mislabeling. Node-support: posterior probabilities (BA) and 1000 bootstrap pseudo-replicates (ML/MP). Scale-bars: substitutions/site (BA and ML) and number of substitutions (MP).

**Additional file 6: Figure S4.** Depth-coverage for full-breadth-coverage consensus sequences in which ≥1 position had depth-coverage <10 reads. Red: sequences with mean depth-coverage <10 reads/position (regarded as unreliable and excluded from phylogenetic analyses); orange: sequences with mean depth-coverage ≥10 reads/position, but with ≥15% of positions supported by <10 reads (regarded as dubious); green: sequences with only short stretches (<15% of sequence length) with depth-coverage was <10 reads (reliable). Y-axes on a log10 scale.

## Data Availability

Data supporting the conclusions of this article are included in the article and its additional files. The newly generated sequences were submitted to the GenBank database under the accession numbers MK411269-MK411278.

## Abbreviations

*AmpG*: amplicon G (a putative nuclear intron)
BA: Bayesian (phylogenetic) analyses
BIC: Bayesian information criterion
CNRS: Centre National de la Recherche Scientifique
*cytb*: mitochondrial *cytochrome b* gene
D2-*28S*: D2 variable region of the *28S* of the nuclear rDNA
EMBL-EBI: European Molecular Biology Laboratory, European Bioinformatics Institute
ENA: European Nucleotide Archive
ITS2: second internal transcribed spacer of the nuclear rDNA
ML: maximum likelihood
MP: maximum parsimony
NCBI: National Center for Biotechnology Information
rDNA: ribosomal DNA
SRA: Sequence Read Archive
T3p: Tamura three-parameter model of nucleotide substitution
UNESP: Universidade Estadual Paulista ‘Júlio de Mesquita Filho’
