# Supplementary figures and images for: Transcriptome-based molecular systematics: Rhodnius montenegrensis (Triatominae) and its position within the Rhodnius prolixus–Rhodnius robustus cryptic–species complex
Source: Parasit Vectors. 2019 Jun 17;12:305. doi: 10.1186/s13071-019-3558-9 (PMC6580618; doi:10.1186/s13071-019-3558-9)

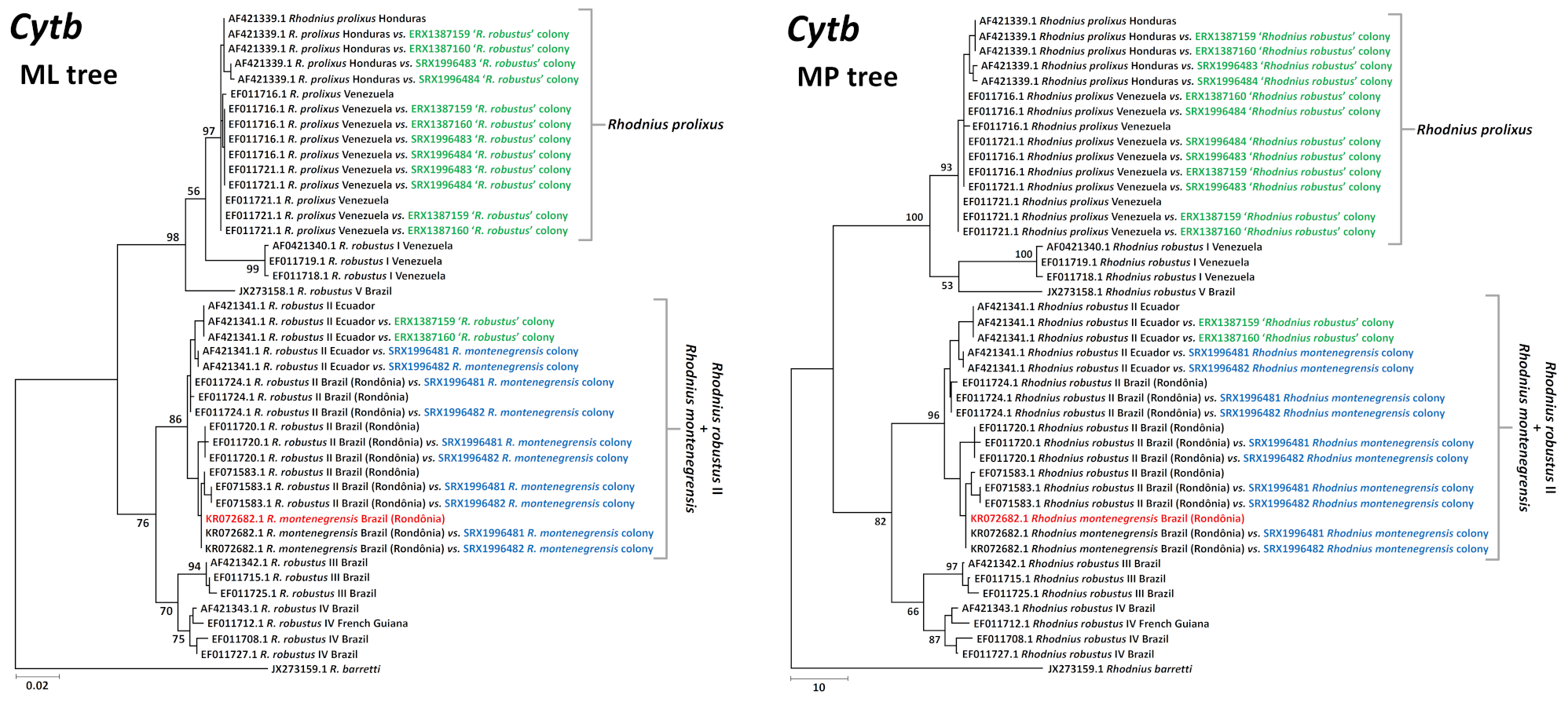

Supplement: Supplementary file 3 — Additional file 3: Figure S1. Cytb trees of members of the Rhodnius prolixus-R. robustus complex. Maximum-likelihood (ML; Tamura 3-parameter+γ) and maximum-parsimony (MP) trees using query sequences (black) and consensus sequences from R. montenegrensis (black/blue) and R. robustus (black/green) transcriptome-read archives (with NCBI codes). Rhodnius montenegrensis’ original sequence (KR072682.1; [7]) highlighted in red. Consensus sequences with mean depth-coverage <10 reads/position were excluded (Table 4, Additional file 6: Figure S4). Node-support: 1000 bootstrap pseudo-replicates. Scale-bars: substitutions/site (ML) and number of substitutions (MP). [file 13071_2019_3558_MOESM3_ESM.tif]

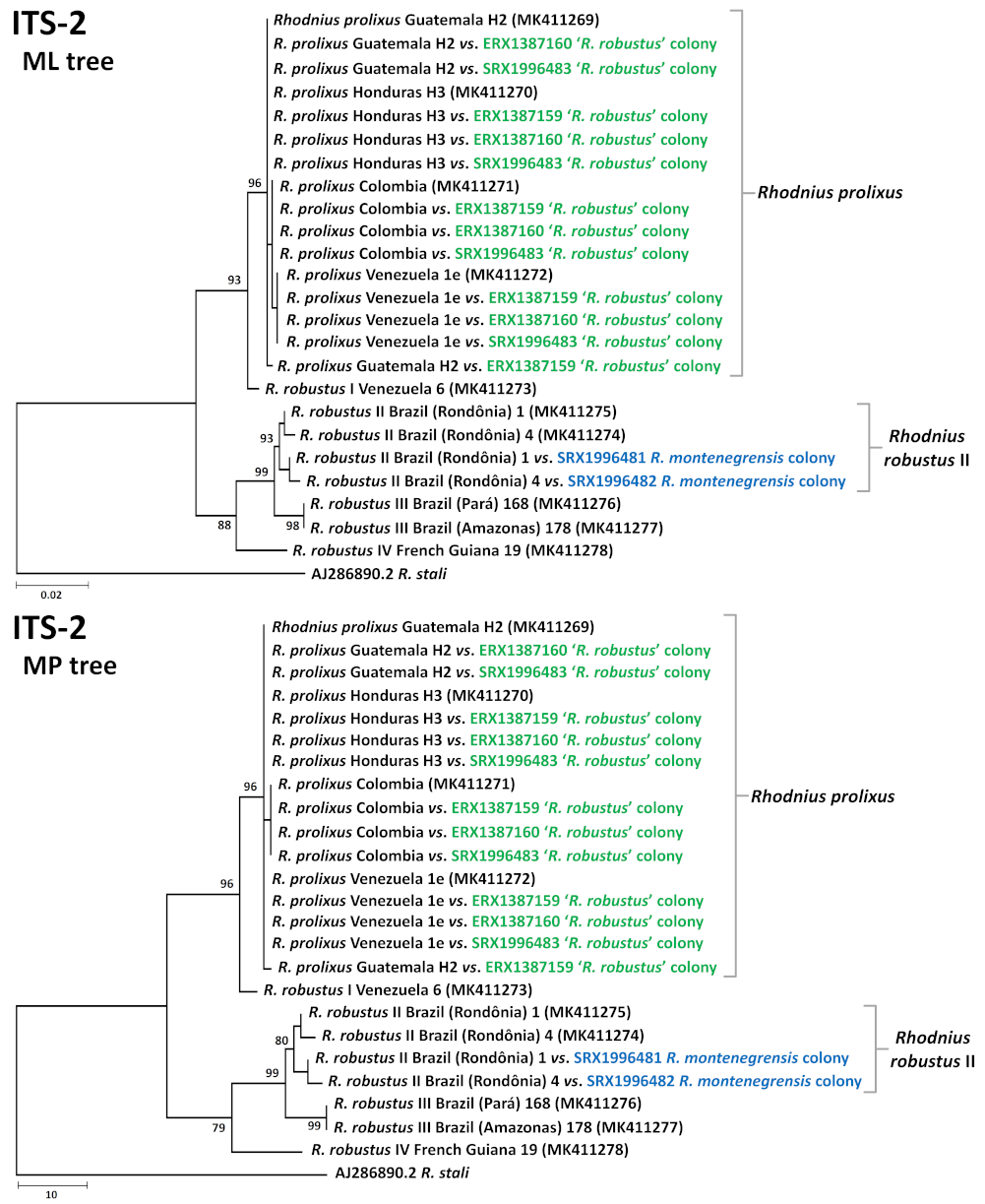

Supplement: Supplementary file 4 — Additional file 4: Figure S2. ITS2 trees of members of the Rhodnius prolixus-R. robustus complex. Maximum-likelihood (ML; Tamura three-parameter) and maximum-parsimony (MP) inferred using query sequences (black) and consensus sequences from R. montenegrensis (black/blue) and R. robustus (black/green) transcriptome-read archives (with NCBI codes). Node-support: 1000 bootstrap pseudo-replicates. Scale-bars: substitutions/site (ML) and number of substitutions (MP). [file 13071_2019_3558_MOESM4_ESM.tif]

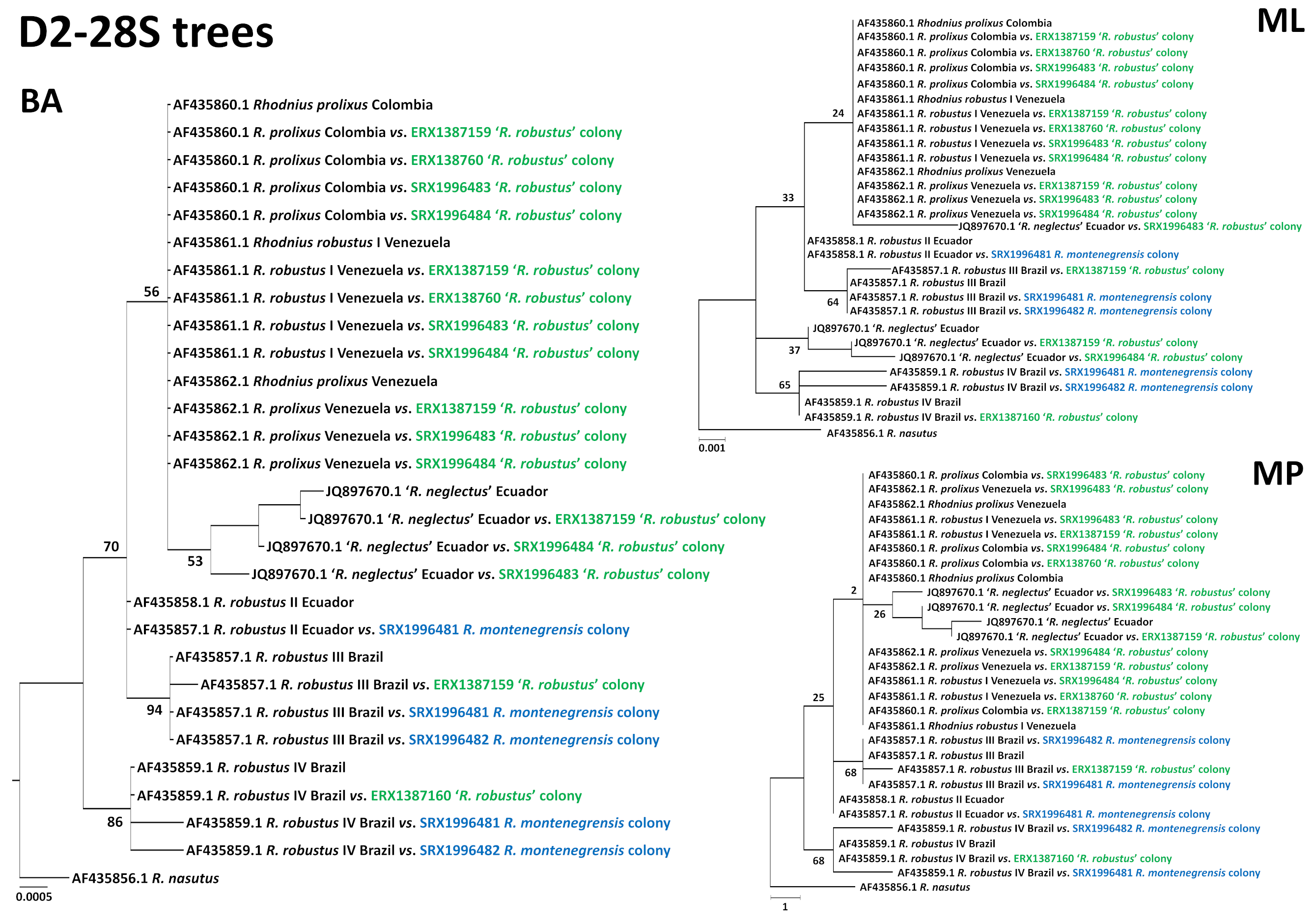

Supplement: Supplementary file 5 — Additional file 5: Figure S3. D2-28S phylogenetic trees of members of the Rhodnius prolixus-R. robustus complex and related taxa. Bayesian analysis (BA), maximum-likelihood (ML; Tamura 3-parameter+γ) and maximum parsimony (MP) trees based on query sequences (black) and consensus sequences from R. montenegrensis and R. robustus transcriptome-read archives (with NCBI codes). JQ897670.1 is from a bug identified as ‘Rhodnius neglectus’ from ‘Orellana, Ecuador’ [31], where R. neglectus does not occur; most likely misidentification/mislabeling. Node-support: posterior probabilities (BA) and 1000 bootstrap pseudo-replicates (ML/MP). Scale-bars: substitutions/site (BA and ML) and number of substitutions (MP). [file 13071_2019_3558_MOESM5_ESM.tif]
