# Supplementary material for: Transcriptome-based molecular systematics: Rhodnius montenegrensis (Triatominae) and its position within the Rhodnius prolixus–Rhodnius robustus cryptic–species complex
Source: Parasit Vectors. 2019 Jun 17;12:305. doi: 10.1186/s13071-019-3558-9 (PMC6580618; doi:10.1186/s13071-019-3558-9)
Supplement: Supplementary file 6 — Additional file 6: Figure S4. Depth-coverage for full-breadth-coverage consensus sequences in which ≥1 position had depth-coverage <10 reads. Red: sequences with mean depth-coverage <10 reads/position (regarded as unreliable and excluded from phylogenetic analyses); orange: sequences with mean depth-coverage ≥10 reads/position, but with ≥15% of positions supported by <10 reads (regarded as dubious); green: sequences with only short stretches (<15% of sequence length) with depth-coverage was <10 reads (reliable). Y-axes on a log10 scale. [file 13071_2019_3558_MOESM6_ESM.pdf]

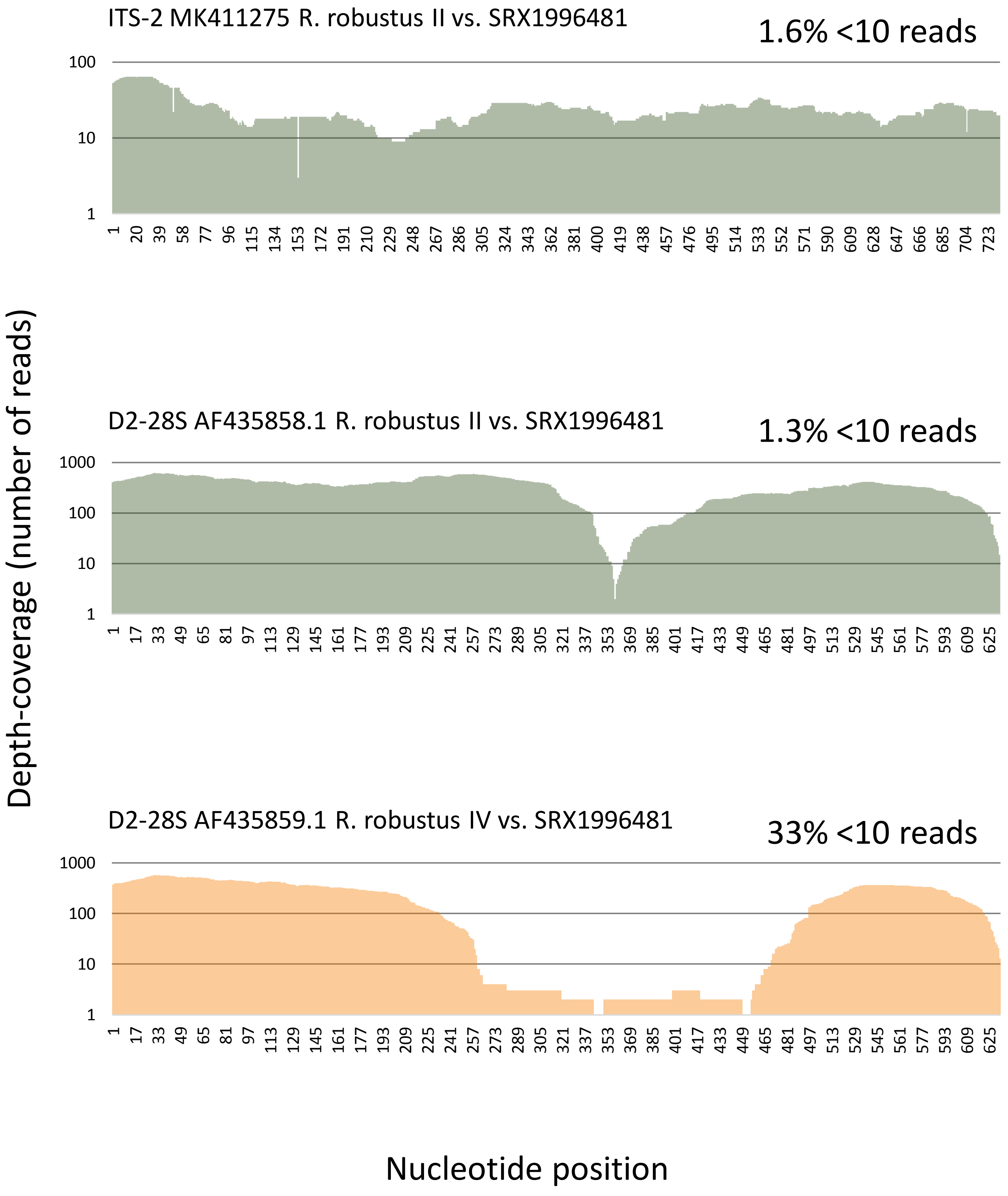

ITS-2 MK411275 R. robustus II vs. SRX1996481
1.6% <10 reads
D2-28S AF435858.1 R. robustus II vs. SRX1996481
1.3% <10 reads
D2-28S AF435859.1 R. robustus IV vs. SRX1996481
33% <10 reads
Depth-coverage (number of reads)
Nucleotide position

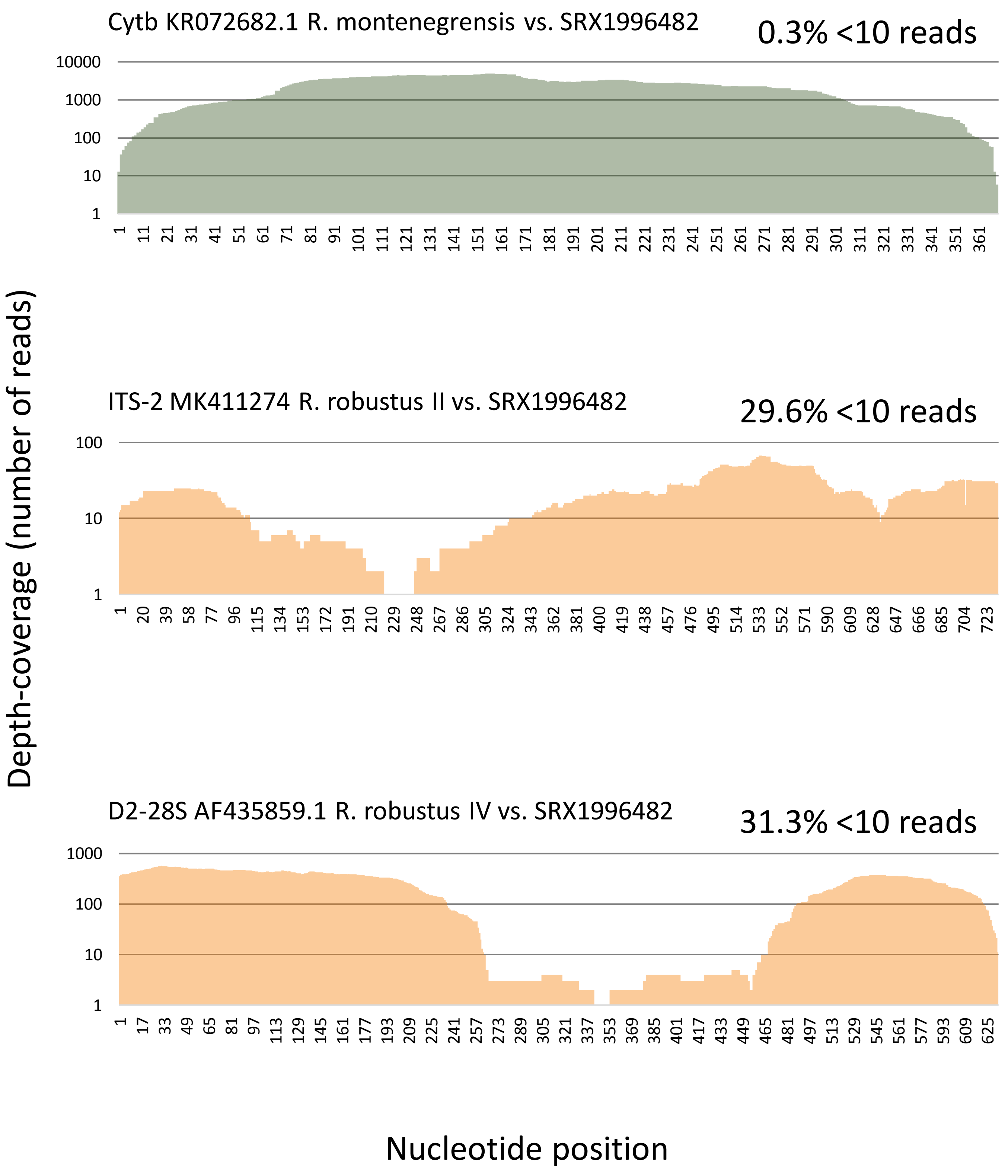
Cytb KR072682.1 R. montenegrensis vs. SRX1996482
0.3% <10 reads
ITS-2 MK411274 R. robustus II vs. SRX1996482
29.6% <10 reads
D2-28S AF435859.1 R. robustus IV vs. SRX1996482
31.3% <10 reads
Depth-coverage (number of reads)
Nucleotide position

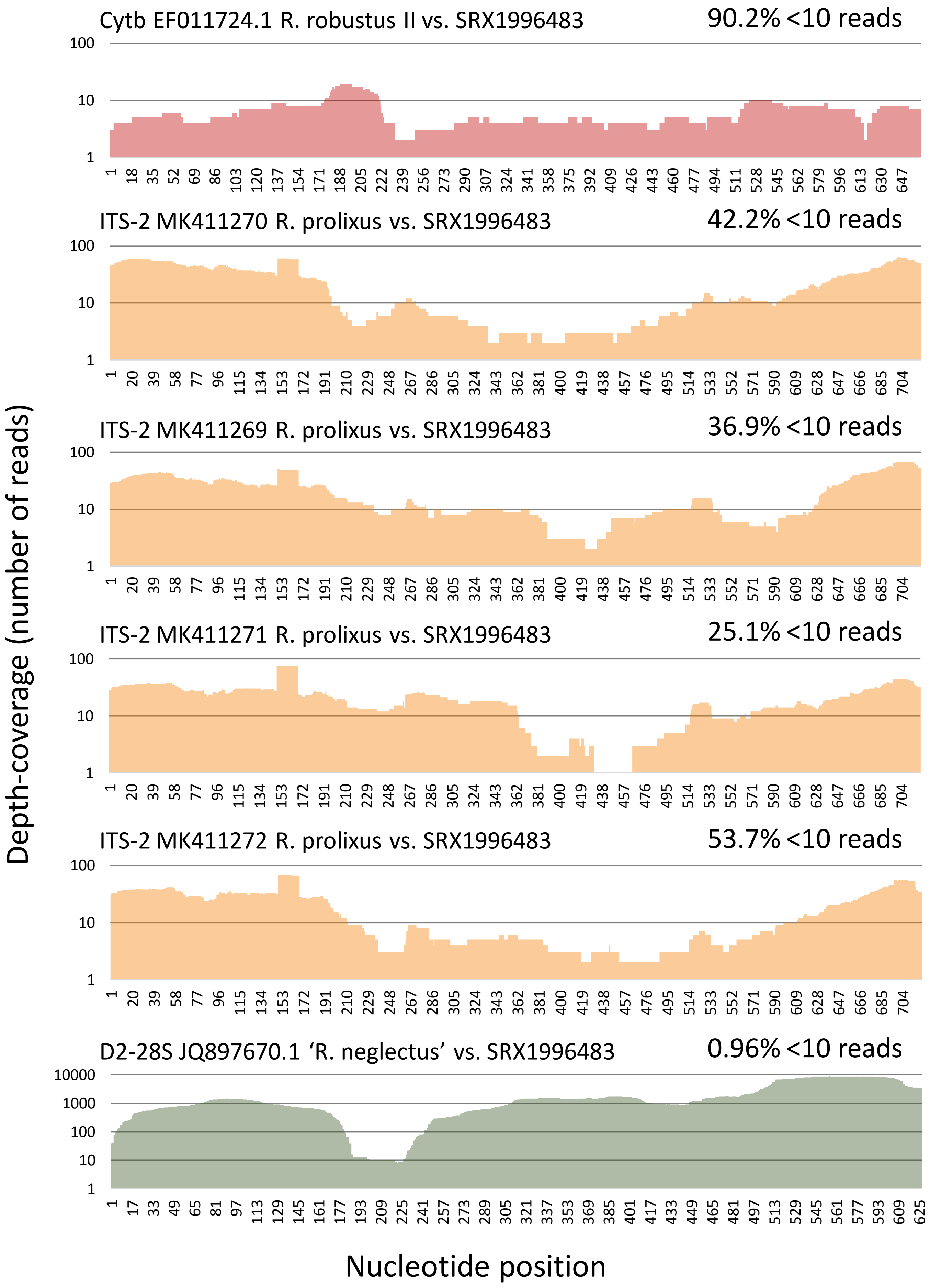

Cytb EF011724.1 R. robustus II vs. SRX1996483
90.2% <10 reads
ITS-2 MK411270 R. prolixus vs. SRX1996483
42.2% <10 reads
ITS-2 MK411269 R. prolixus vs. SRX1996483
36.9% <10 reads
ITS-2 MK411271 R. prolixus vs. SRX1996483
25.1% <10 reads
ITS-2 MK411272 R. prolixus vs. SRX1996483
53.7% <10 reads
D2-28S JQ897670.1 'R. neglectus' vs. SRX1996483
0.96% <10 reads
Depth-coverage (number of reads)
Nucleotide position

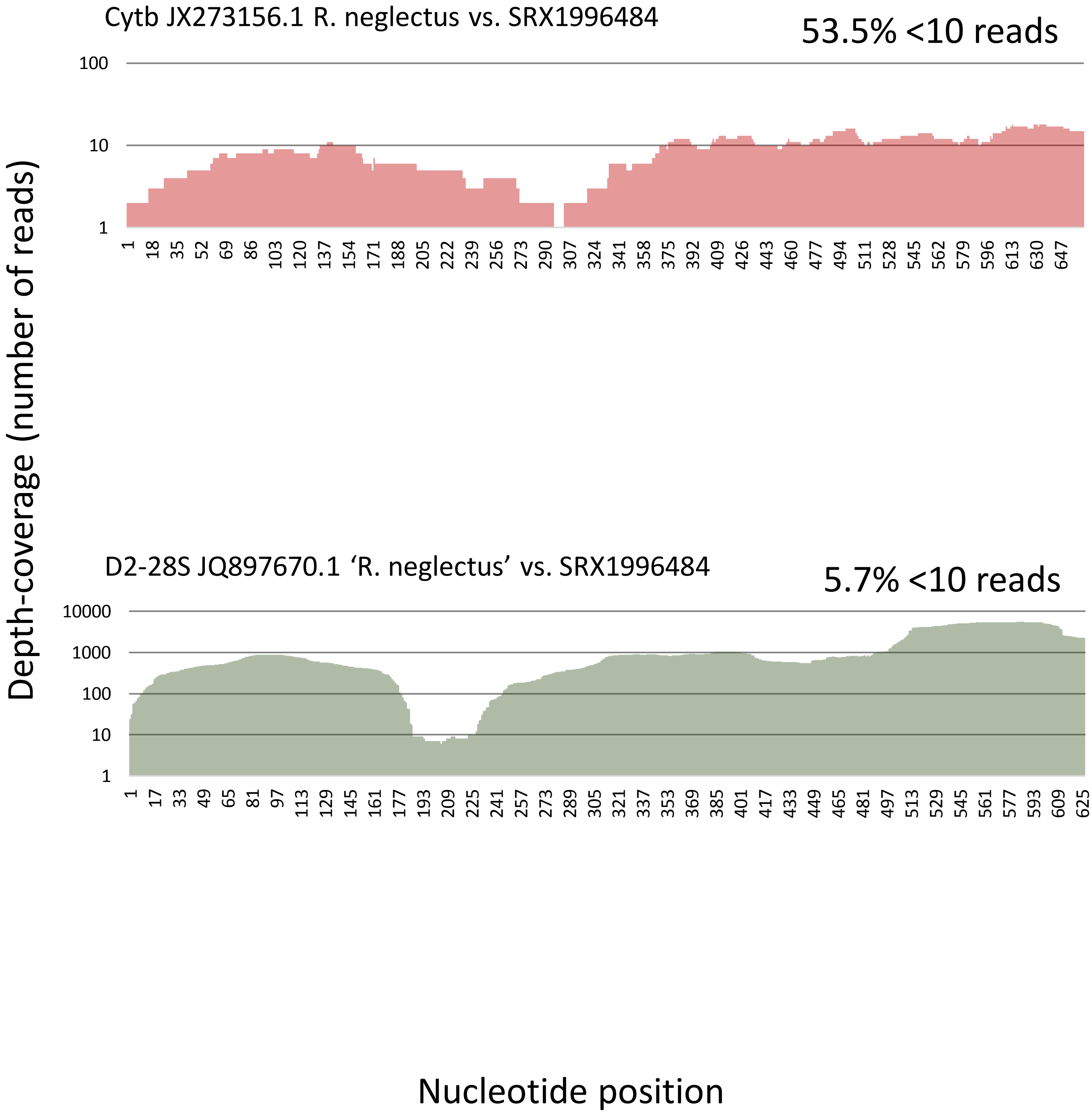

Cytb JX273156.1 R. neglectus vs. SRX1996484
53.5% <10 reads
100
10
1
1 18 35 52 69 86 103 120 137 154 171 188 205 222 239 256 273 290 307 324 341 358 375 392 409 426 443 460 477 494 511 528 545 562 579 596 613 630 647
D2-28S JQ897670.1 'R. neglectus' vs. SRX1996484
5.7% <10 reads
10000
1000
100
10
1
1 17 33 49 65 81 97 113 129 145 161 177 193 209 225 241 257 273 289 305 321 337 353 369 385 401 417 433 449 465 481 497 513 529 545 561 577 593 609 625
Depth-coverage (number of reads)
Nucleotide position

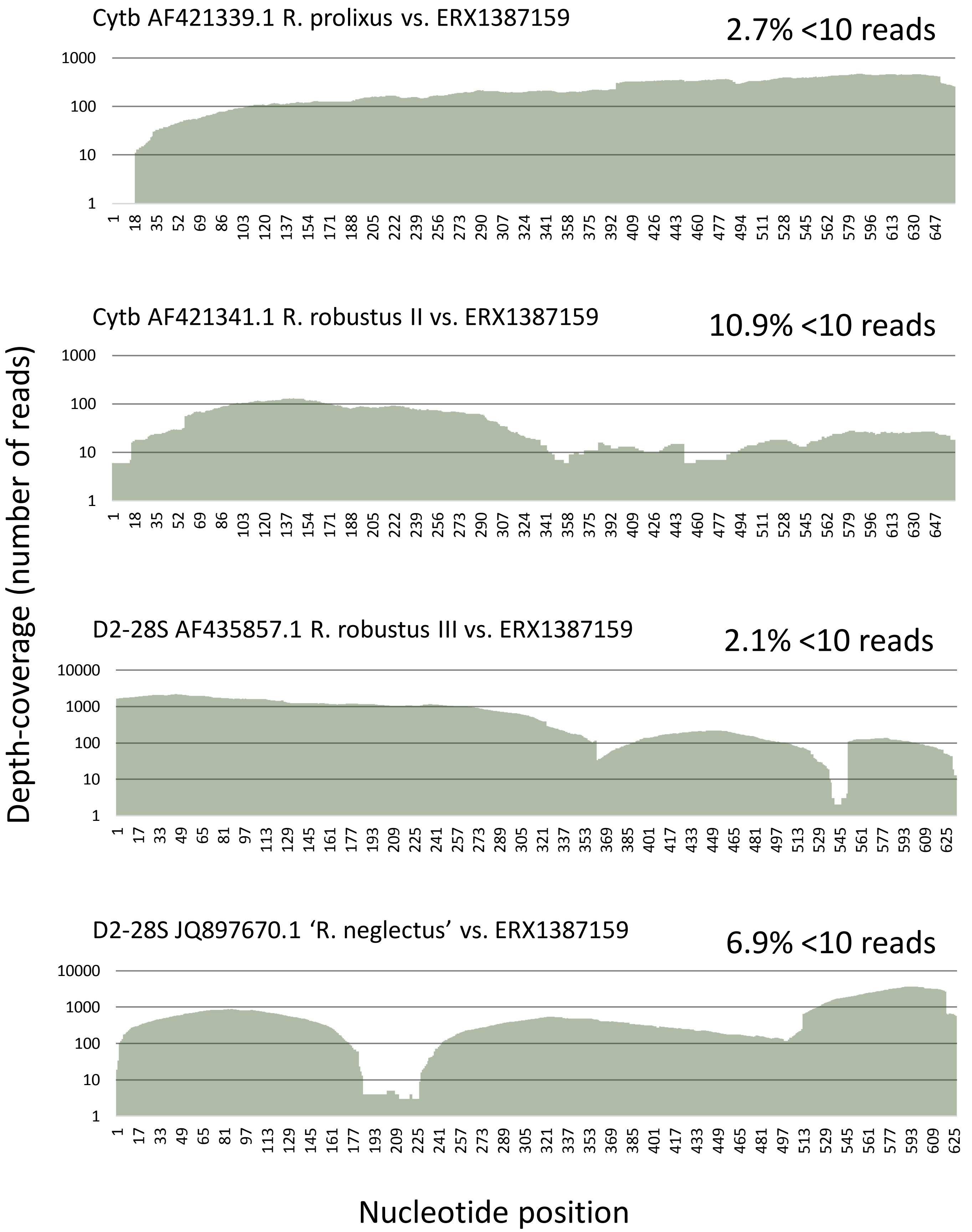
Cytb AF421339.1 R. prolixus vs. ERX1387159
2.7% <10 reads
Cytb AF421341.1 R. robustus II vs. ERX1387159
10.9% <10 reads
D2-28S AF435857.1 R. robustus III vs. ERX1387159
2.1% <10 reads
D2-28S JQ897670.1 'R. neglectus' vs. ERX1387159
6.9% <10 reads
Depth-coverage (number of reads)
Nucleotide position

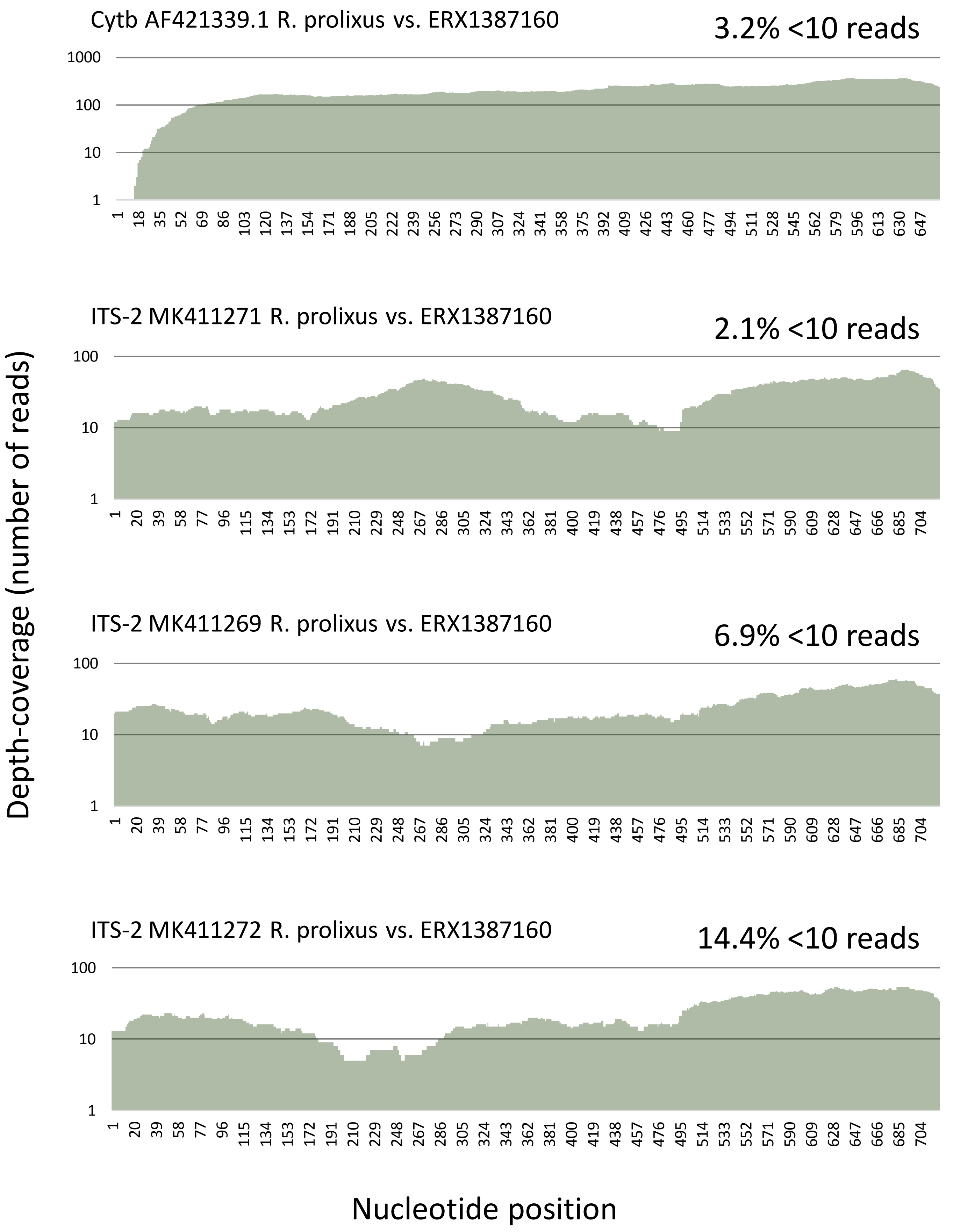

Cytb AF421339.1 R. prolixus vs. ERX1387160
3.2% <10 reads
1000
100
10
1
1 18 35 52 69 86 103 120 137 154 171 188 205 222 239 256 273 290 307 324 341 358 375 392 409 426 443 460 477 494 511 528 545 562 579 596 613 630 647
ITS-2 MK411271 R. prolixus vs. ERX1387160
2.1% <10 reads
100
10
1
1 20 39 58 77 96 115 134 153 172 191 210 229 248 267 286 305 324 343 362 381 400 419 438 457 476 495 514 533 552 571 590 609 628 647 666 685 704
ITS-2 MK411269 R. prolixus vs. ERX1387160
6.9% <10 reads
100
10
1
1 20 39 58 77 96 115 134 153 172 191 210 229 248 267 286 305 324 343 362 381 400 419 438 457 476 495 514 533 552 571 590 609 628 647 666 685 704
ITS-2 MK411272 R. prolixus vs. ERX1387160
14.4% <10 reads
100
10
1
1 20 39 58 77 96 115 134 153 172 191 210 229 248 267 286 305 324 343 362 381 400 419 438 457 476 495 514 533 552 571 590 609 628 647 666 685 704
Depth-coverage (number of reads)
Nucleotide position

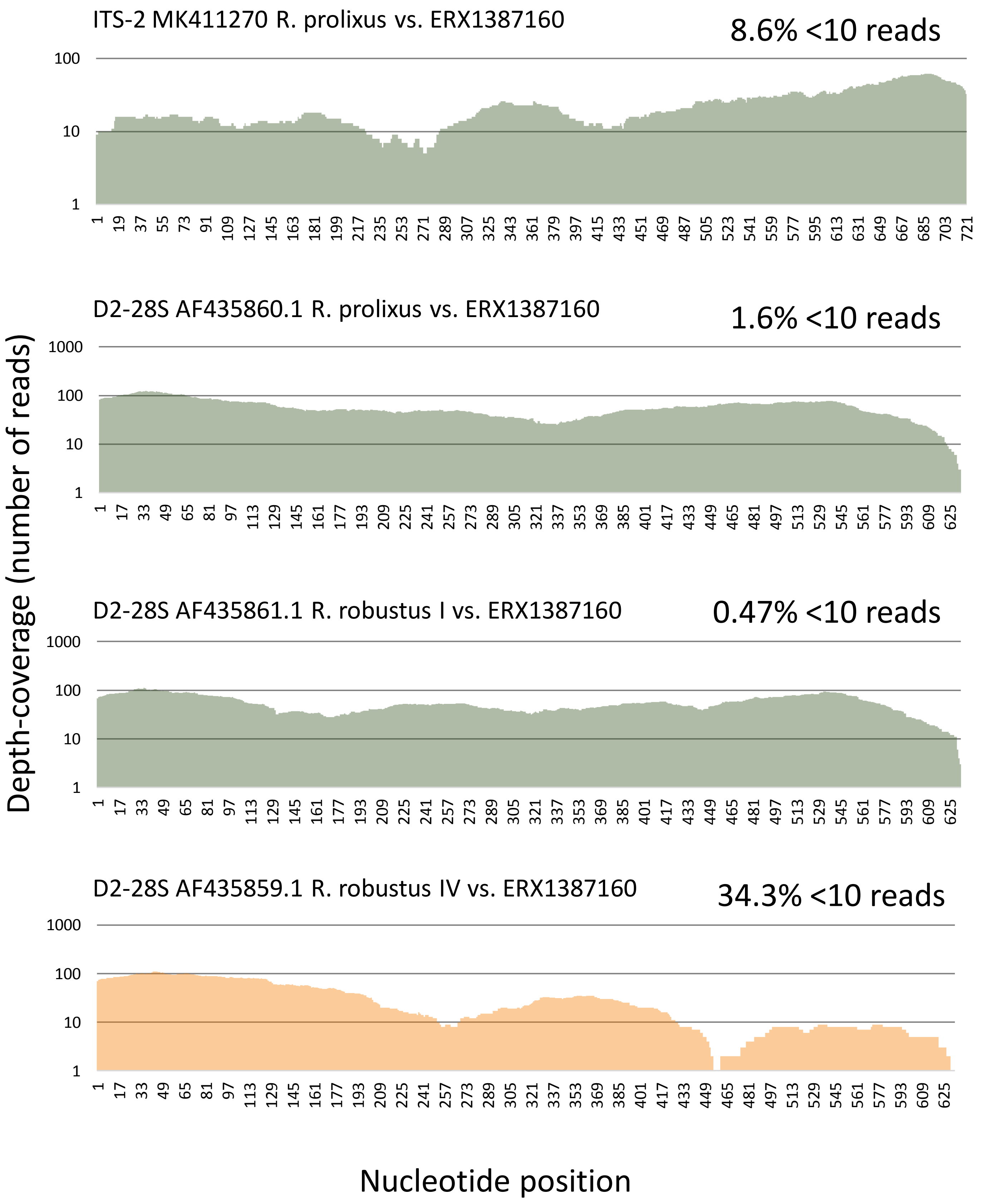
ITS-2 MK411270 R. prolixus vs. ERX1387160
8.6% <10 reads
100
10
1
1 19 37 55 73 91 109 127 145 163 181 199 217 235 253 271 289 307 325 343 361 379 397 415 433 451 469 487 505 523 541 559 577 595 613 631 649 667 685 703 721
D2-28S AF435860.1 R. prolixus vs. ERX1387160
1.6% <10 reads
1000
100
10
1
1 17 33 49 65 81 97 113 129 145 161 177 193 209 225 241 257 273 289 305 321 337 353 369 385 401 417 433 449 465 481 497 513 529 545 561 577 593 609 625
D2-28S AF435861.1 R. robustus I vs. ERX1387160
0.47% <10 reads
1000
100
10
1
1 17 33 49 65 81 97 113 129 145 161 177 193 209 225 241 257 273 289 305 321 337 353 369 385 401 417 433 449 465 481 497 513 529 545 561 577 593 609 625
D2-28S AF435859.1 R. robustus IV vs. ERX1387160
34.3% <10 reads
1000
100
10
1
1 17 33 49 65 81 97 113 129 145 161 177 193 209 225 241 257 273 289 305 321 337 353 369 385 401 417 433 449 465 481 497 513 529 545 561 577 593 609 625
Depth-coverage (number of reads)
Nucleotide position
